# Metabolic profiles of *Sri Lankan cassava mosaic virus*-infected and healthy cassava (*Manihot esculenta* Crantz) cultivars with tolerance and susceptibility phenotypes

**DOI:** 10.1186/s12870-023-04181-3

**Published:** 2023-04-05

**Authors:** Somruthai Chaowongdee, Srihunsa Malichan, Pornkanok Pongpamorn, Atchara Paemanee, Wanwisa Siriwan

**Affiliations:** 1 Center for Agricultural Biotechnology, Kasetsart University, Kamphaeng Saen Campus, Nakhon Pathom, 73140 Thailand; 2 Center of Excellence on Agricultural Biotechnology (AG-BIO/MHESI), Bangkok, 10900 Thailand; 3 Department of Plant Pathology, Faculty of Agriculture, Kasetsart University, Bangkok, 10900 Thailand; 4 National Omics Center (NOC), National Science and Technology Development Agency (NSTDA), Pathum Thani, 12120 Thailand

**Keywords:** Cassava, Metabolite profile, *Sri Lankan Cassava Mosaic Virus*, Tolerant and susceptible

## Abstract

**Background:**

Cassava mosaic disease (CMD) of cassava (*Manihot esculenta* Crantz) has expanded across many continents. *Sri Lankan cassava mosaic virus* (SLCMV; family *Geminiviridae*), which is the predominant cause of CMD in Thailand, has caused agricultural and economic damage in many Southeast Asia countries such as Vietnam, Loas, and Cambodia. The recent SLCMV epidemic in Thailand was commonly found in cassava plantations. Current understanding of plant–virus interactions for SLCMV and cassava is limited. Accordingly, this study explored the metabolic profiles of SLCMV-infected and healthy groups of tolerant (TME3 and KU50) and susceptible (R11) cultivars of cassava. Findings from the study may help to improve cassava breeding, particularly when combined with future transcriptomic and proteomic research.

**Results:**

SLCMV-infected and healthy leaves were subjected to metabolite extraction followed by ultra-high-performance liquid chromatography high-resolution mass spectrometry (UHPLC-HRMS/MS). The resulting data were analyzed using Compound Discoverer software, the mzCloud, mzVault, and ChemSpider databases, and published literature. Of the 85 differential compounds (SLCMV-infected vs healthy groups), 54 were differential compounds in all three cultivars. These compounds were analyzed using principal component analysis (PCA), hierarchical clustering dendrogram analysis, heatmap analysis, and Kyoto Encyclopedia of Genes and Genomes (KEGG) pathway annotation. Chlorogenic acid, DL-carnitine, neochlorogenic acid, (E)-aconitic acid, and ascorbyl glucoside were differentially expressed only in TME3 and KU50, with chlorogenic acid, (E)-aconitic acid, and neochlorogenic acid being downregulated in both SLCMV-infected TME3 and KU50, DL-carnitine being upregulated in both SLCMV-infected TME3 and KU50, and ascorbyl glucoside being downregulated in SLCMV-infected TME3 but upregulated in SLCMV-infected KU50. Furthermore, 7-hydroxycoumarine was differentially expressed only in TME3 and R11, while quercitrin, guanine, N-acetylornithine, uridine, vorinostat, sucrose, and lotaustralin were differentially expressed only in KU50 and R11.

**Conclusions:**

Metabolic profiling of three cassava landrace cultivars (TME3, KU50, and R11) was performed after SLCMV infection and the profiles were compared with those of healthy samples. Certain differential compounds (SLCMV-infected vs healthy groups) in different cultivars of cassava may be involved in plant–virus interactions and could underlie the tolerance and susceptible responses in this important crop.

**Supplementary Information:**

The online version contains supplementary material available at 10.1186/s12870-023-04181-3.

## Background

Cassava (*Manihot esculenta* Crantz) is the third most common plant-based carbohydrate source after rice and maize [1]. It is used for human consumption, animal feed, and plant-based energy production. More than 800 million people use cassava as their main food crop in Africa, Asia, and Latin America [2]. In Thailand in 2016, the cassava product capacity was 30.5 million tons/year, the mean yield was 0.56 tons/hectare, and 20–25% of the product was used to meet domestic demand rather than export [3]. In 2019, Thailand provided 79.56% of the global exported cassava, making it the world’s largest cassava exporter [4, 5].

In Southeast Asia, the majority of lost cassava yield is due to cassava mosaic disease (CMD), which is caused by *Cassava mosaic virus* (CMV; genus *Begomovirus*; family *Geminiviridae*) [2, 6, 7]. CMV can be transmitted from external sources to nearby fields by whitefly (*Bemisia tabaci* Gennadius) and infected propagative materials [2, 5, 6]. CMV comprises 11 species, nine of which are found in Africa [8, 9], while there are two main species in Asia: *Sri Lankan cassava mosaic virus* (SLCMV) and *Indian cassava mosaic virus* (ICMV) [10]. Recently, SLCMV has caused CMD outbreaks in Cambodia [11], Vietnam [12], Thailand [13], and Laos [14]. Plantations with documented CMV acted as the origin of CMV that was subsequently transmitted to nearby countries or provinces. Consequently, understanding plant–virus interactions and developing a new SLCMV-resistant cultivar are key topics of current research on SLCMV.

Metabolomics analyses based on mass spectrometry (MS) have been used to investigate the mechanisms of viral infection resistance and tolerance in plants [15, 16]. Primary and secondary metabolites are instrumental in plant immune responses, including physiological and biochemical disease resistance responses [17]. Differences in metabolomic profiles can provide an explanation for different viral infection responses, with plant genotypic and corresponding phenotypic variations altering disease severity [18]. There are three phenotypes of viral infection responses in plants: (1) resistance, where the plant exhibits resistance gene expression that completely restricts virus multiplication and therefore there are no disease symptoms [19]; (2) tolerance, involving recovery after disease symptoms in a plant that exhibits adaptations to the viral infection [20], with reductions in both disease symptoms and virus titers [21]; and (3) susceptibility, where the virus multiplies in the plant and then systemically expands throughout the plant [20]. These different plant phenotypes lead to unique plant–virus interactions, involving metabolic (including protein) changes [17, 22, 23]*.*

To date, only four geminivirus resistance genes (*R*-genes) in crops have been found, mapped, cloned, and studied [24–27], indicating that a relatively small amount of the genetic diversity naturally present for geminivirus disease resistance has been utilized. In cassava, three known genetic resistance loci found in the germplasm provide CMD with a comparatively stable field resistance. *CMD1* is a recessive locus that was introgressed from wild cassava [28], the single dominant gene locus *CMD2* in tropical *Manihot esculenta* (TME) cultivars confers resistance to all known CMVs [29, 30], and *CMD3* can be distinguished from *CMD2* which applied to be a single marker [31]. Although the underlying molecular mechanism and robustness of the *CMD2* locus are currently unknown, this locus is the predominant resistance source used in African cassava breeding programs because a single dominant gene greatly facilitates breeding. Further research is needed on resistance genes/markers in cassava, but *CMD2* represents a reasonable explanation for the differential tolerance of TME3 vs KU50, which are two cultivars of cassava [32, 33]. *CMD2* in TME3 (tolerant cultivar) may underlie the key cassava–SLCMV interactions that lead to CMD tolerance.

To understand more about *CMD2*, Kuon et al. (2019) [34] reported the long-based de novo assembled genomes of CMD-susceptible and *CMD2*-resistant African cassava cultivars, and the assembled results facilitated genetic mapping approaches to narrow the large *CMD2* region to a few candidate genes that might explain the development of robust CMD resistance. Recently, Lim et al. (2022) [35] found that CMD2-type landraces that have undergone regeneration through de novo morphogenesis lose their resistance. The CMD2 locus was fine mapped to a 190-kb interval because whole genome sequencing and genetic variant analysis after full genome bisulfite sequencing failed to identify an epigenetic mechanism for this loss of resistance. These findings suggest that a nonsynonymous single nucleotide polymorphism in *DNA polymerase subunit 1* (*MePOLD1*), which is present in this region, is the source of CMD2-type resistance. Furthermore, virus-induced *MePOLD1* gene silencing in a CMD2-type resistance-prone cassava variety led to a recovery phenotype.

Metabolomics analysis is a type of omics that is crucial for studying plant resistance and susceptibility. There are some reports of the primary or specialized metabolism of a plant changing in response to a plant pathogen. Agustika et al*.* [36] examined compounds from *Pepper yellow leaf curl virus* (PYLCV)-infected and healthy chili using a principal component analysis (PCA) of gas chromatography-mass spectrometry (GC–MS) data. The first three principal components (PCs) explained 91.32% of the variance among PYLCV-infected and healthy chili, distinguishing between the metabolic patterns of PYLCV-infected and non-infected chili. Furthermore, GC–MS has been used to investigate volatile organic compound release by infected plants such as citrus plants, lettuce (*Lactuca sativa* L.), maize, and sweet cherry (*Prunusavium* L.) [37–39]. This procedure has also been used to explore powdery mildew of sugar beet [40] and *Fusarium coeruleum*-infected potato [41]. Meanwhile, recent research involving metabolomics investigation of *Phytophthora sojae*-infected soybeans revealed a number of sugars and secondary metabolites that were differentially increased in resistant plants compared with the susceptible plants, suggesting that these compounds may be involved in defense mechanisms in plants [42]. Traditional approaches to study plant disease have relied on phenotypic analyses, including comparisons of symptom development between susceptible and resistant varieties, and various molecular readouts of plant defense mechanisms. Consequently, metabolomics research has been highlighted as an important tool to expand knowledge of plant-pathogen interactions and further the understanding of metabolites within plant defense mechanisms [43].

The main objective of this study was to compare the metabolic profiles of SLCMV-infected tolerant and susceptible cassava cultivars. Three cassava landrace cultivars were selected: TME3, Kasetsart 50 (KU50), and Rayong-11 (R11). The former two cultivars represent tolerant cultivars; TME3 has been reported and cultivated as a CMD-tolerant cultivar for many region expanded the Africa continent [10, 29, 44], while KU50 was mentioned as a CMD-tolerant cultivar in Thailand and neighboring countries in South-East Asia [5, 45]. The remaining cultivar, R11, represents a susceptible cultivar. Findings from the study improve our understanding of plant–virus interactions (in particular, metabolic changes in the host plant after infection), including providing a simplified overview of the phenotypic variation in response to viral infection [19, 20, 46]. This basic knowledge of metabolic mechanisms can help to inform future research, such as that on resistance genes, loci, and metabolite markers, in order to develop a SLCMV-resistant cultivar.

## Results

### SLCMV symptoms and PCR confirmation

SLCMV infection caused various severities of CMD symptoms in the plants included in this study. Cultivars TME3 and KU50 had mild symptoms, with mosaic chorosis and a few abnormal leaves (small leaflet size and distortion), and the two cultivars showed no difference in severity based on visual symptoms. R11 exhibited mosaic chlorosis (with neither light green nor yellow patterns), abnormal leaves, and some twig stunting in the propagated cassava. Thus, although there were marked differences between SLCMV-infected and healthy groups for all three cultivars, the severity differed between cultivars (Additional Fig. 1). Next, SLCMV-infected and healthy leaves were subjected to PCR using *AV1* gene-specific primers [29]. As expected, the amplified fragment size from SLCMV-infected leaves were approximately 928 bp (Additional Fig. 2).

**Fig. 1:**
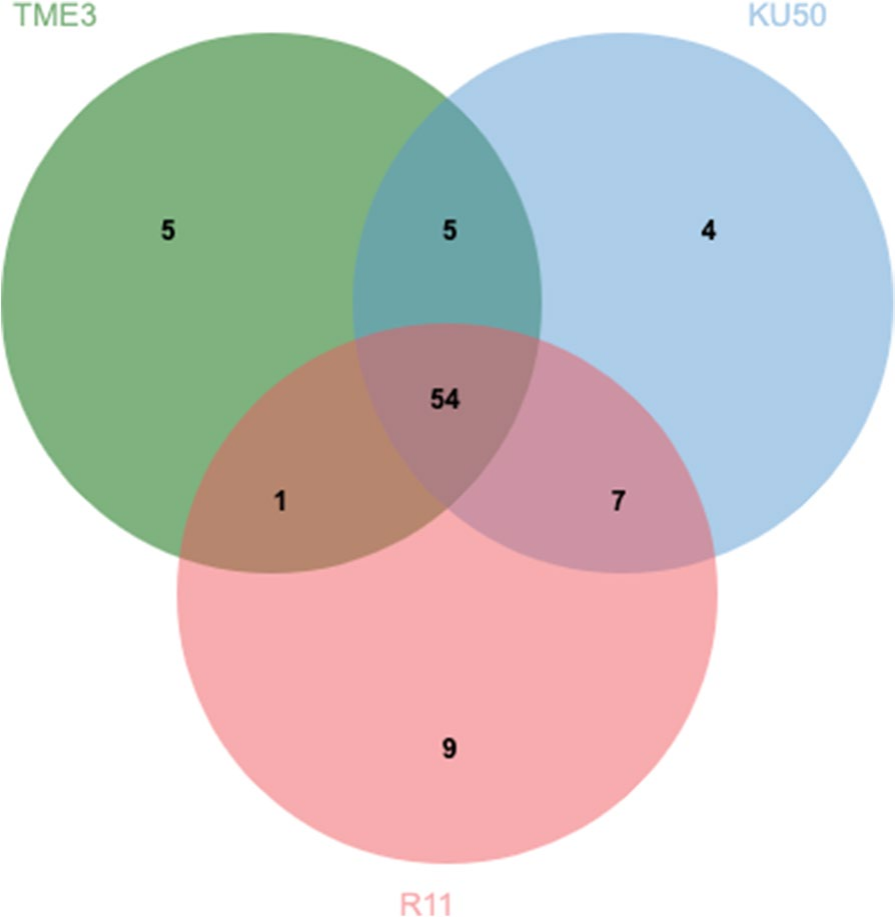
Venn diagram of differential compounds (healthy vs SLCMV-infected groups), using UHPLC-HRMS/MS data, for TME3, KU50, and R11. The green circle represents total compounds found in TME3 cultivar, the blue circle represents the total compounds found in KU50 cultivar, and the red circle represents the total compounds found in R11 cultivar

**Fig. 2:**
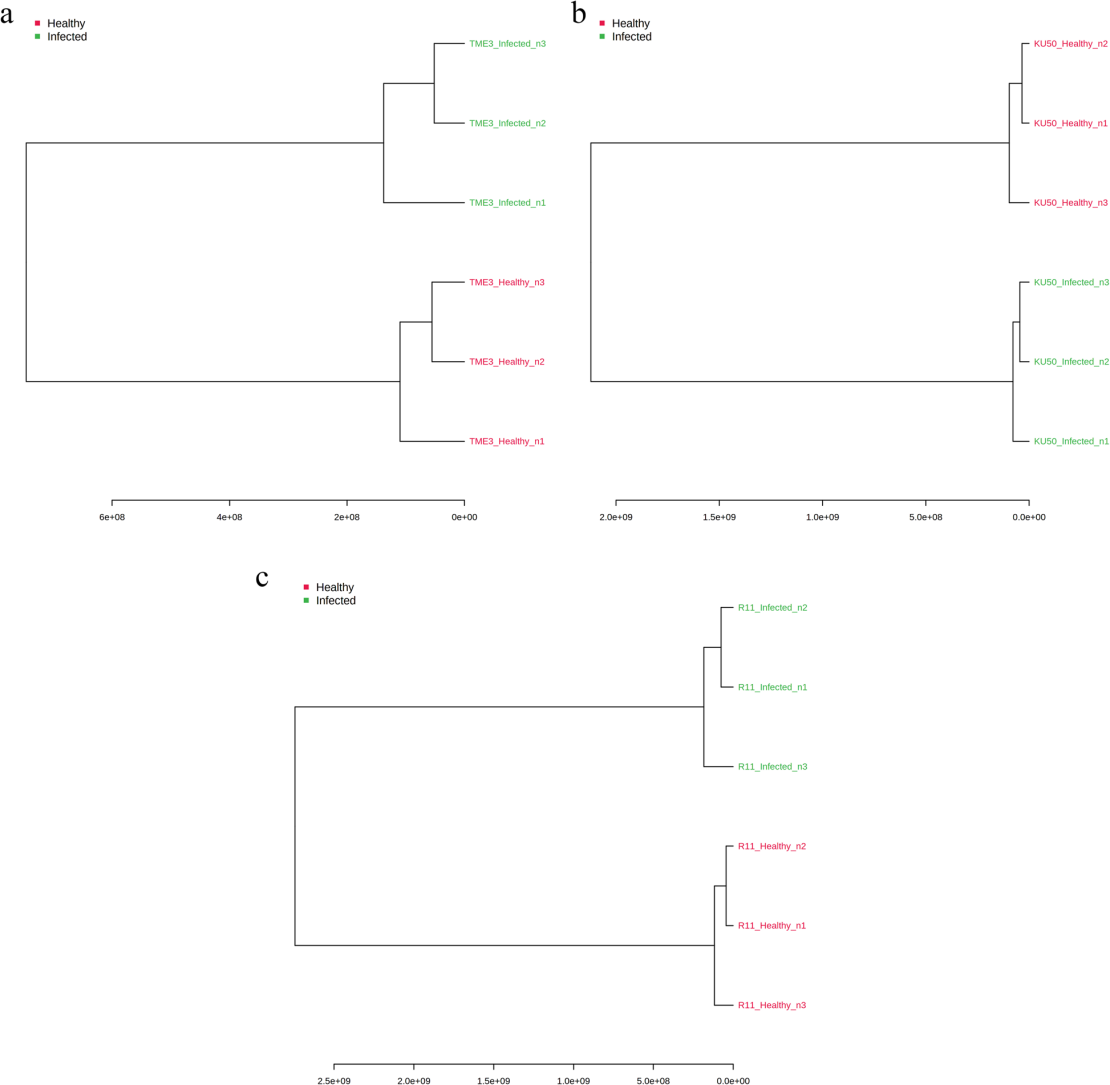
Hierarchical clustering dendrograms of differential compounds (healthy vs SLCMV-infected groups) for (**a**) TME3 (**b**) KU50, and (**c**) R11 cultivars individually, based on Ward clustering algorithm and Euclidean distance metric

### Differential compounds (SLCMV-infected vs healthy samples) in each cultivar

For each of the three cultivars (representing three genotypes), ultra-high-performance liquid chromatography high-resolution mass spectrometry (UHPLC-HRMS/MS) data revealed the identity of differential compounds between the healthy and SLCMV-infected groups.

A total of 85 differential compounds were identified, including proteins, sugars, sugar phosphates, amino acids, organic acids, and starch (Additional file 1). These 85 compounds underwent Kyoto Encyclopedia of Genes and Genomes (KEGG) pathway annotation, based on *Arabidopsis thaliana* (model organism) and cassava databases. A total of 40 pathways were identified through this process (Table 1), and numerous compounds were involved in secondary metabolism such as phenylpropanoid, flavonoid, and ubiquinone biosynthesis*.*

**Table 1:** 85 differential compounds (healthy vs SLCMV-infected groups) identified by UHPLC-HRMS/MS and annotated in KEGG pathway analysis. Total is the total number of detected compounds which matched in the pathway; Hits is the number of differential compounds in the pathway; Raw *P* is the original *P* value calculated in the enrichment analysis; Holm *P* is the *P* value adjusted by the Holm-Bonferroni method; FDR *P* is the *P* value adjusted using the false discovery rate; Impact is the pathway impact value calculated using pathway topology analysis

Pathways	KEGG pathway	Total	Expected	Hits	Raw p	log10 (p)	Holm adjust	FDR	Impact
Aminoacyl-tRNA biosynthesis	map00970	46	1.2097	10	9.7818E-08	7.0096	9.3905E-06	9.3905E-06	0.11111
Flavone and flavonol biosynthesis	map00944	10	0.26298	3	0.0017776	2.7502	0.16887	0.068052	0.7
Valine, leucine and isoleucine biosynthesis	map00290	22	0.57856	4	0.0021266	2.6723	0.1999	0.068052	0
Glyoxylate and dicarboxylate metabolism	map00630	29	0.76264	4	0.0060453	2.2186	0.56222	0.14509	0.0781
Alanine, aspartate and glutamate metabolism	map00250	22	0.57856	3	0.018329	1.7369	1	0.35191	0.32374
Flavonoid biosynthesis	map00941	47	1.236	4	0.032308	1.4907	1	0.51693	0.00425
Cyanoamino acid metabolism	ko00460	29	0.76264	3	0.038316	1.4166	1	0.52548	0
Glycine, serine and threonine metabolism	map00260	33	0.86784	3	0.053238	1.2738	1	0.60842	0.30168
Sulfur metabolism	map00920	15	0.39447	2	0.057039	1.2438	1	0.60842	0.03315
Valine, leucine and isoleucine degradation	map00280	37	0.97303	3	0.07057	1.1514	1	0.62395	0
Butanoate metabolism	map00650	17	0.44707	2	0.071494	1.1457	1	0.62395	0
Glucosinolate biosynthesis	map00966	65	1.7094	4	0.087535	1.0578	1	0.70028	0
Citrate cycle (TCA cycle)	map00020	20	0.52596	2	0.095105	1.0218	1	0.70231	0.15581
Phenylalanine, tyrosine and tryptophan biosynthesis	map00400	22	0.57856	2	0.11193	0.95106	1	0.74948	0.02152
Phenylpropanoid biosynthesis	map00940	46	1.2097	3	0.11745	0.93014	1	0.74948	0.07013
Biosynthesis of secondary metabolites—unclassified	map01110	5	0.13149	1	0.12491	0.90339	1	0.74948	1
Glutathione metabolism	map00480	26	0.68375	2	0.14761	0.83087	1	0.78936	0.06248
Isoquinoline alkaloid biosynthesis	map00950	6	0.15779	1	0.148	0.82973	1	0.78936	0.5
Tropane, piperidine and pyridine alkaloid biosynthesis	map00960	8	0.21038	1	0.19242	0.71575	1	0.97222	0
Arginine and proline metabolism	map00330	34	0.89413	2	0.22424	0.64929	1	1	0.07781
Phenylalanine metabolism	map00360	11	0.28928	1	0.25484	0.59373	1	1	0.47059
Ubiquinone and other terpenoid-quinone biosynthesis	map00130	38	0.99933	2	0.26382	0.5787	1	1	0.00097
Nitrogen metabolism	map00910	12	0.31558	1	0.27458	0.56133	1	1	0
Histidine metabolism	map00340	15	0.39447	1	0.3308	0.48043	1	1	0.04264
Tyrosine metabolism	map00350	16	0.42077	1	0.34858	0.4577	1	1	0.10811
Sphingolipid metabolism	map00600	17	0.44707	1	0.3659	0.43664	1	1	0
Ascorbate and aldarate metabolism	map00053	18	0.47336	1	0.38277	0.41707	1	1	0
Lysine degradation	map00310	18	0.47336	1	0.38277	0.41707	1	1	0
Arginine biosynthesis	map00220	18	0.47336	1	0.38277	0.41707	1	1	0.08544
Pentose phosphate pathway	map00030	19	0.49966	1	0.3992	0.39881	1	1	0
Propanoate metabolism	map00640	20	0.52596	1	0.4152	0.38174	1	1	0
Zeatin biosynthesis	map00908	21	0.55226	1	0.43079	0.36573	1	1	0
Starch and sucrose metabolism	map00500	22	0.57856	1	0.44598	0.35069	1	1	0.13619
Pantothenate and CoA biosynthesis	map00770	23	0.60486	1	0.46077	0.33652	1	1	0
Purine metabolism	map00230	63	1.6568	2	0.50037	0.30071	1	1	0.00126
Galactose metabolism	map00052	27	0.71005	1	0.51617	0.28721	1	1	0
Inositol phosphate metabolism	map00562	28	0.73635	1	0.52913	0.27644	1	1	0
Glycerophospholipid metabolism	map00564	37	0.97303	1	0.63153	0.19961	1	1	0.03075
Cysteine and methionine metabolism	map00270	46	1.2097	1	0.7121	0.14746	1	1	0
Porphyrin and chlorophyll metabolism	map00860	48	1.2623	1	0.72752	0.13815	1	1	0

A Venn diagram showed that 54 of the 85 differential compounds were shared by all three cultivars (Fig. 1; Table 2). In addition, five compounds (sphinganine, gentiopicrin, glucogallin, glucoheptonic acid, and nitrosoguvacoline) were uniquely differentially expressed in TME3, four (robinetin, guanosine, linamarin, and uric acid) in KU50, and nine (3-amino-2-naphthoic acid, afzelin, arginine, succinic acid, L-glutathione, prolylleucine, scopoletin, cytidine, and DL-citrulline) in R11. Five compounds (chlorogenic acid, DL-carnitine, neochlorogenic acid, (E)-aconitic acid, and ascorbyl glucoside) were differentially expressed only in TME3 and KU50, one (7-hydroxycoumarine) was differentially expressed in R11 and TME3, and there were seven compounds (quercitrin, guanine, N-acetylornithine, uridine, vorinostat, sucrose, and lotaustralin) that were differentially expressed in both KU50 and R11.

**Table 2:** 54 differential compounds (healthy vs SLCMV-infected groups) in all three cassava cultivars (TME3, KU50, and R11) and annotated in KEGG pathway analysis. The compound labels are from the KEGG database or other references (as mentioned in the table). The table is based on comma separated values (.csv) files. mes- is the KEGG ID for the *Manihot esculenta* (cassava) model organism

Compounds name	KEGG ID and others	Metabolic pathways	*p*.value	FDR	-log10(p)
p-coumaric acid	C00811	-Ubiquinone and other terpenoid-quinone biosynthesis, Tyrosine metabolism and	9.8287E-21	5.3075E-19	20.008
		-Biosynthesis of phenylpropanoids			
Ferulic acid	mesc01100	-Phenylpropanoid biosynthesis^c^	1.0714E-18	2.8928E-17	17.97
L-Phenylalanine	C00079, mesc00960 and mesc00460	-Tropane, piperidine and pyridine alkaloid biosynthesis. -Cyanoamino acid metabolism	2.809E-18	5.0562E-17	17.551
Caffeic acid	C01197	-Phenylpropanoid biosynthesis and Biosynthesis of phenylpropanoids	5.4956E-18	7.4191E-17	17.26
Kaempferol	C05903 and mesc00941	-Flavonoid biosynthesis	1.8769E-16	1.9135E-15	15.727
DL-Tryptophan	C00078	-Phenylalanine, tyrosine, and tryptophan biosynthesis	2.1261E-16	1.9135E-15	15.672
		-Aminoacyl-tRNA biosynthesis			
		-Glycine, serine, and threonine metabolism			
L-Asparagine	C00152 and mesc01230	-Biosynthesis of amino acids	8.2644E-16	6.3754E-15	15.083
DL-Arginine	C00062 and mesc01230	-Biosynthesis of amino acids	1.5382E-15	9.7835E-15	14.813
L-(-)-Serine	C00065 and mesc01230	-Biosynthesis of amino acids	1.6306E-15	9.7835E-15	14.788
L-Histidine	C00135, mesc01230 and mesc02010	-Biosynthesis of amino acids and ABC transporters	2.7682E-15	1.3869E-14	14.558
trans-3-Indoleacrylic acid	PubChemCID5375048^a^	-Tryptophan metabolism	2.8252E-15	1.3869E-14	14.549
L-Leucine	C00123 and mesc01230	-Biosynthesis of amino acids	9.1396E-15	4.1128E-14	14.039
DL-Glutamine	PubChemCID738^a^	-Peptidoglycan cytoplasmic synthesis and recycling pathways	2.8017E-14	1.1638E-13	13.553
Myricetin	C10107, mesc00944 and mesc00941	-Flavonoid biosynthesis	7.9059E-14	3.0494E-13	13.102
Rutin	C05625, mesc00944 and mesc00941	-Flavone and flavonol biosynthesis	1.2223E-13	4.4001E-13	12.913
L-Isoleucine	C00407 and mesc01230	-Biosynthesis of amino acids	2.1915E-13	6.9614E-13	12.659
L-Pyroglutamic acid	C01879	-Glutathione metabolism	2.8449E-13	8.5348E-13	12.546
Hyperoside	C10073	-Flavone and flavonol biosynthesis	5.1965E-13	1.4769E-12	12.284
Kaempferol-3-O-rutinoside	C21833	-Flavone and flavonol biosynthesis	6.6022E-13	1.7826E-12	12.18
Tran-ferulic acid	-	-Phenylpropanoids^b^	7.7382E-13	1.9898E-12	12.111
Trifolin	mesc00944	-Flavone and flavonol biosynthesis	9.7426E-13	2.3914E-12	12.011
Mauritianin	C10178	-Flavone and flavonol biosynthesis	1.5242E-12	3.5786E-12	11.817
Rhamnetin	C10176	-Flavone and flavonol biosynthesis	1.8069E-12	4.0655E-12	11.743
Adenine	C00147 and mesc01232	-Nucleotide metabolism	2.2669E-12	4.8964E-12	11.645
D-(-)-Aspartic acid	C00402	-Biosynthesis of amino acids	2.7206E-12	5.6437E-12	11.565
Gallic acid	C01424	-Aminobenzoate degradation and biosynthesis of phenylpropanoids	2.8218E-12	5.6437E-12	11.549
DL-Lysine	PubChemCID866^a^	- Amino acid and derivatives^b^	5.0739E-12	9.7854E-12	11.295
Adenosine	C00212 and mesc01232	-Nucleotide metabolism	6.2633E-12	1.1663E-11	11.203
Citric acid	C00158	-Glutamate metabolism and Citrate cycle (TCA cycle) and Alanine aspartate	1.3109E-11	2.3597E-11	10.882
Quercetin	C00389	-Flavone and flavonol biosynthesis	1.537E-11	2.6773E-11	10.813
L-Threonine	C00188, mesc00290 and mesc01230	-Biosynthesis of amino acids, valine, leucine, and isoleucine biosynthesis	2.6953E-11	4.5483E-11	10.569
N-Acetylmuramic acid	C02713	-Biosynthesis of nucleotide sugars and Phosphotransferase system (PTS) and Amino sugar and nucleotide sugar metabolism	4.9555E-11	8.1091E-11	10.305
L-Glutamic acid	C00025	-Alanine, proline, aspartate, and glutamate metabolism Aminoacyl-tRNA biosynthesis	5.7894E-11	9.195E-11	10.237
L-Tyrosine	C00082, mesc00970, mesc00360 and mesc00730	- Aminoacyl-tRNA biosynthesis and Phenylalanine metabolism andThiamine metabolism	6.344E-11	9.7879E-11	10.198
Pipecolic acid	C00408	- Lysine degradation and Tropane and Piperidine and pyridine alkaloid biosynthesis	9.1537E-11	1.373E-10	10.038
4-Guanidinobutyric acid	C01035	-Arginine and proline metabolism	1.6724E-10	2.4407E-10	9.7767
Luteolin	C01514 and mesc00941	-Flavone and flavonol biosynthesis	4.6938E-10	6.6702E-10	9.3285
Valine	C00183, mesc00290 and mesc00280	-Valine, leucine and isoleucine biosynthesis and degradation	1.6357E-09	2.2285E-09	8.7863
Choline	C00114, mesc02010 and mesc00260	-ABC transporters and Glycine, serine, and threonine metabolism	1.6508E-09	2.2285E-09	8.7823
Trigonelline	mesc00960	-Tropane, piperidine and pyridine alkaloid biosynthesis	3.6166E-09	4.7634E-09	8.4417
D-(-)-Quinic acid	C00296	-Phenylalanine, tyrosine, and tryptophan biosynthesis	1.6833E-08	2.1643E-08	7.7738
Tricine	PubChemCID79784^a^	Other	4.0104E-08	5.0363E-08	7.3968
Vitamin C	C00072	- Ascorbate and aldarate metabolism^d,c^ and Glutathione metabolism	2.3659E-07	2.9036E-07	6.626
(2R)-dihomocitric acid	C16583, mjv01210 and mjv01240	- 2-Oxocarboxylic acid metabolism and Biosynthesis of cofactors	1.4856E-06	1.7827E-06	5.8281
D-Glucose 6-phosphate	C00092 and mesc00500	-Starch and sucrose metabolism	2.6351E-06	3.0934E-06	5.5792
APM	HMDB0034252^b^	-Organic acids and derivatives	4.9873E-06	5.7301E-06	5.3021
D-( +)-Glucose	PubChemCID107526^a^	-Sugar^b^	8.5747E-06	9.6465E-06	5.0668
3-Methylinosine	PubChemCID126961054^a^	Others	9.8133E-06	1.0815E-05	5.0082
Furamizole	C14304	Others	0.0001501	0.0001609	3.8236
Gluconic acid	C00257	- Pentose phosphate pathway and Carbon metabolism	0.00015196	0.0001609	3.8183
D-Glucaric acid	C00818	- Ascorbate and aldarate metabolism and Cell wall precursor/ sugar acid^a^	0.00096612	0.00098435	3.015
Hexose	C00738	-Cellular hexose transport^b^	0.0010293	0.0010293	2.9874

^a^PubChem CID; PubChem (https://pubchem.ncbi.nlm.nih.gov/), National Center for Biotechnology Information (2022). PubChem Patent Summary for CN-107849574-B. Retrieved August 27, 2022 from https://pubchem.ncbi.nlm.nih.gov/patent/CN-107849574-B

^b^HMDB ID; Wishart DS, Tzur D, Knox C, et al., *HMDB: the Human Metabolome Database.* Nucleic Acids Res. 2007 Jan;35(Database issue):D521-6. 17,202,168 (https://hmdb.ca/)

^c^Drapal M., Barros de Carvalho E., Ovalle Rivera T. M., Becerra Lopez-Lavalle L. A., and Fraser P. D., 2019. Capturing Biochemical Diversity in Cassava (*Manihot esculenta* Crantz) through the Application of Metabolite Profiling. *Journal of Agricultural and Food Chemistry* 2019 *67* (3), 986–993. https://doi.org/10.1021/acs.jafc.8b04769

^d^Hori H. Methylated nucleosides in tRNA and tRNA methyltransferases. Front Genet. 2014 May 23;5:144. https://doi.org/10.3389/fgene.2014.00144. PMID: 24,904,644; PMCID: PMC4033218

**Fig. 3:**
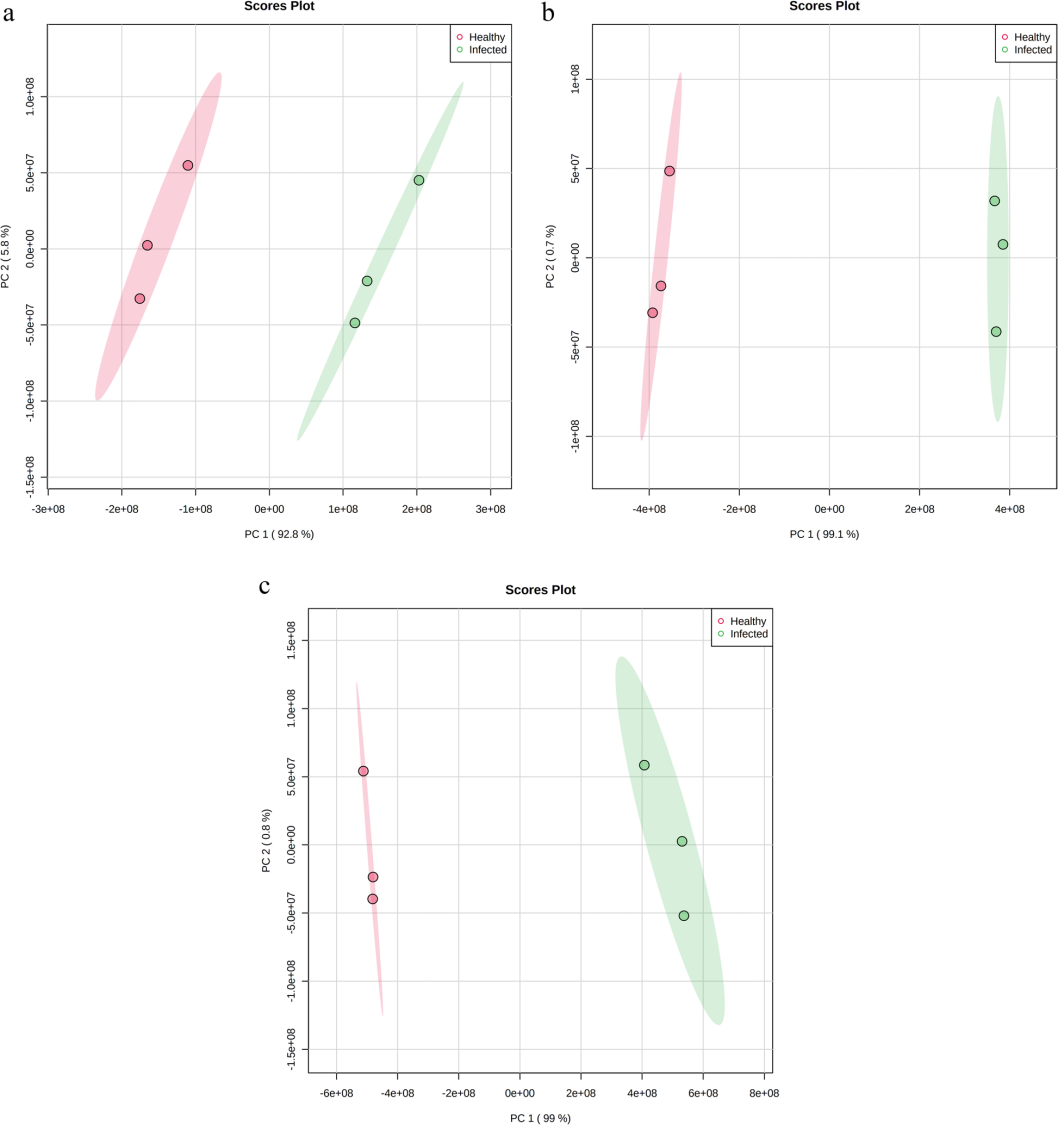
2D principal component analysis (PCA) score plots of differential compounds (SLCMV-infected vs healthy groups) for (**a**) TME3 (**b**) KU50, and (**c**) R11 cultivars individually

Hierarchical clustering dendrograms, based on 65, 70, and 71 differential compounds for TME3, KU50, and R11, respectively, showed that there was clear separation of SLCMV-infected and healthy groups for the three cultivars (Fig. 2). 2D PCA score plots also showed that the SLCMV-infected group was clearly separated from the healthy group for each of the three cultivars (Fig. 3), with PC1 explaining 92.8% of the variance and PC2 explaining 5.8% for TME3, PC1 and PC2 explaining 99.1% and 0.7%, respectively, for KU50, and PC1 and PC2 respectively explaining 99% and 0.8% of the variance for R11 (Fig. 3).

**Fig. 4:**
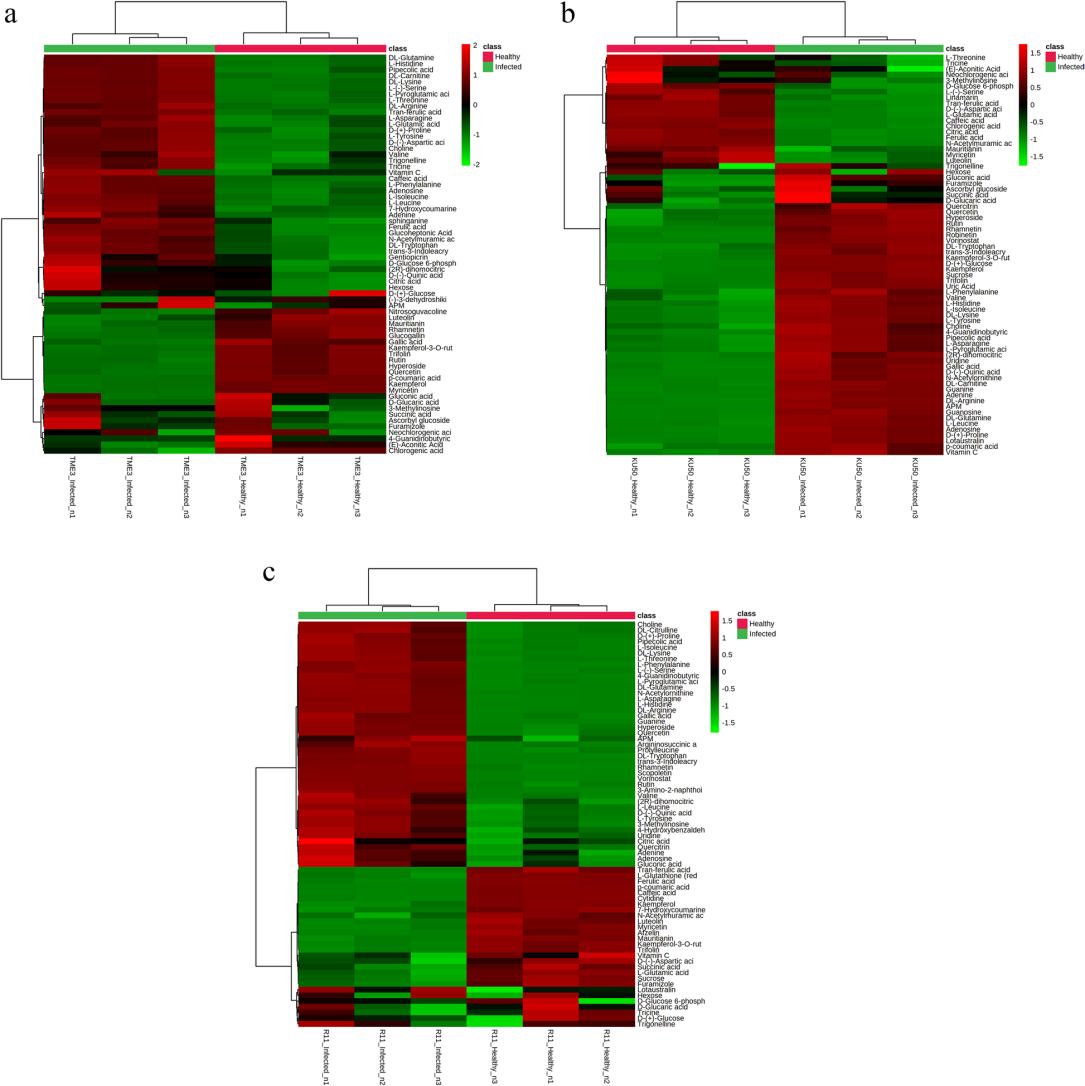
Heatmaps of differential compounds (healthy vs SLCMV-infected groups) for (**a**) TME3 (**b**) KU50, and (**c**) R11 cultivars individually, based on Ward clustering method with Euclidean distance metric and one-way ANOVA followed by Fisher’s LSD (*P* < 0.05). Red represents upregulated compounds and green represents downregulated compounds

Furthermore, detailed examination of the specific cassava cultivars was conducted using heatmaps. Commencing with the TME3 cultivar (Fig. 4a), was divided into two main sections—one that comprised 24 features (compounds) in the lower part of the heatmap that were predominantly associated with the healthy condition, and a second section comprising 41 features that were positively associated with the SLCMV-infected group rather than healthy cassava. Nitrosoguvacoline, which was especially found in the TME3 cultivar, was classified as a member that was positively detected in the healthy condition. Secondly, for the KU50 cultivar, 19 compounds were grouped as a positive intensity in the healthy cluster, while 51 compounds were identified as positive intensity in the SLCMV-infected KU50 cluster (Fig. 4b). For the R11 cultivar, there were 28 features that exhibited positive intensity in a cluster of healthy R11, and other features exhibited positive intensity in a cluster of SLCMV-infected R11 (Fig. 4c).

### Differential compounds (SLCMV-infected vs healthy samples) in all three cultivars

The 54 compounds found in all three cultivars were analyzed using a hierarchical clustering dendrogram analysis, PCA, and heatmap analysis.

**Fig. 5:**
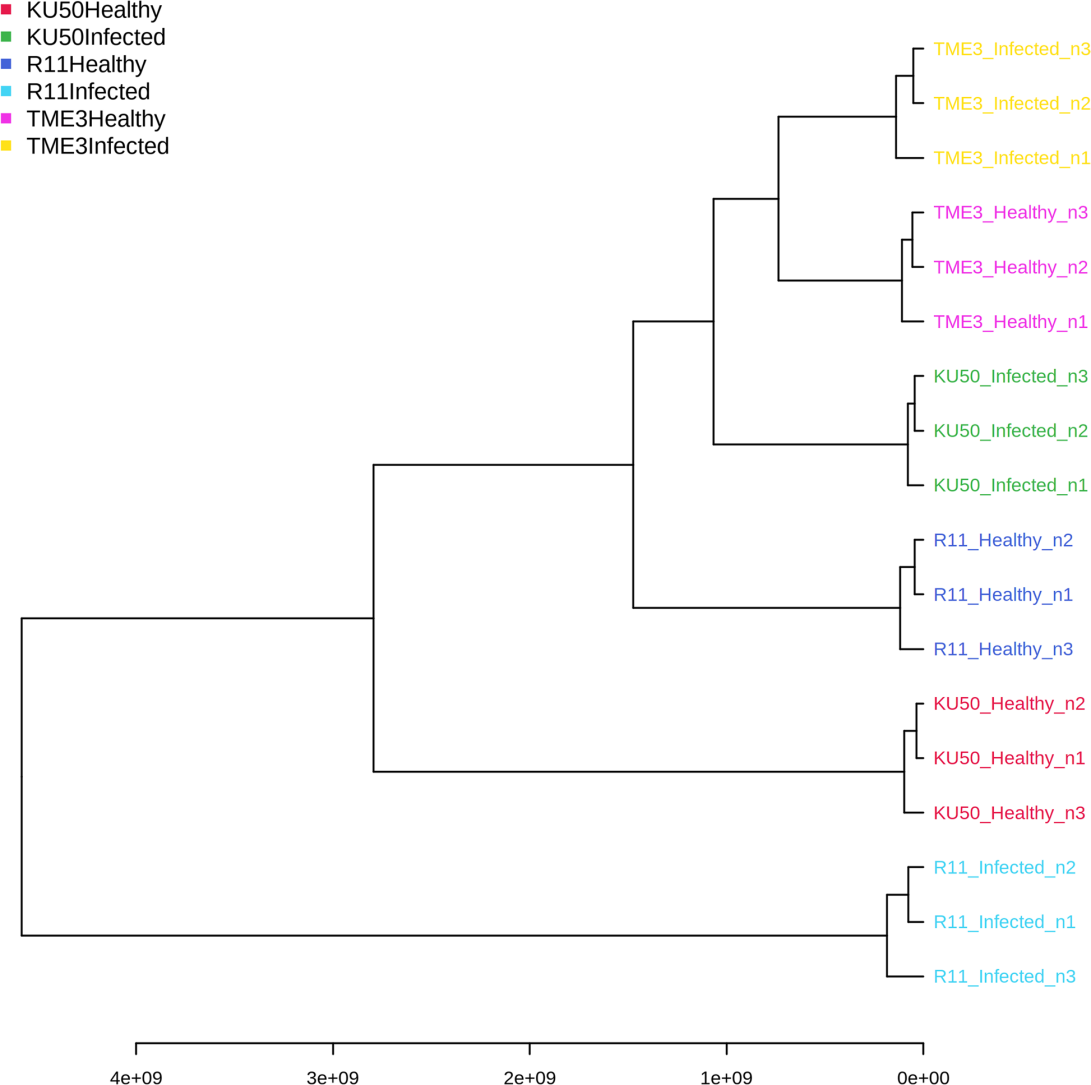
Hierarchical clustering dendrogram of 54 differential compounds (SLCMV-infected vs healthy groups) in all three cultivars (TME3, KU50, and R11), based on Ward clustering algorithm and Euclidean distance metric

Regarding the hierarchical clustering dendrogram based on the 54 compounds in the six experimental groups (Fig. 5), the groups were clustered using Euclidean distance and the Ward clustering algorithm, and then visualized in a dendrogram. This dendrogram featured two main groups (1, healthy TME3, SLCMV-infected TME3, healthy KU50, SLCMV-infected KU50, and healthy R11; and 2, SLCMV-infected R11) and six minor groups (one for each experimental group). SLCMV-infected R11 was clearly separated from the other groups (including healthy R11), which suggests that healthy and SLCMV-infected R11 are relatively unrelated. Healthy and SLCMV-infected TME3 and SLCMV-infected KU50 were clustered together, while healthy and SLCMV-infected KU50 were relatively separate.

**Fig. 6:**
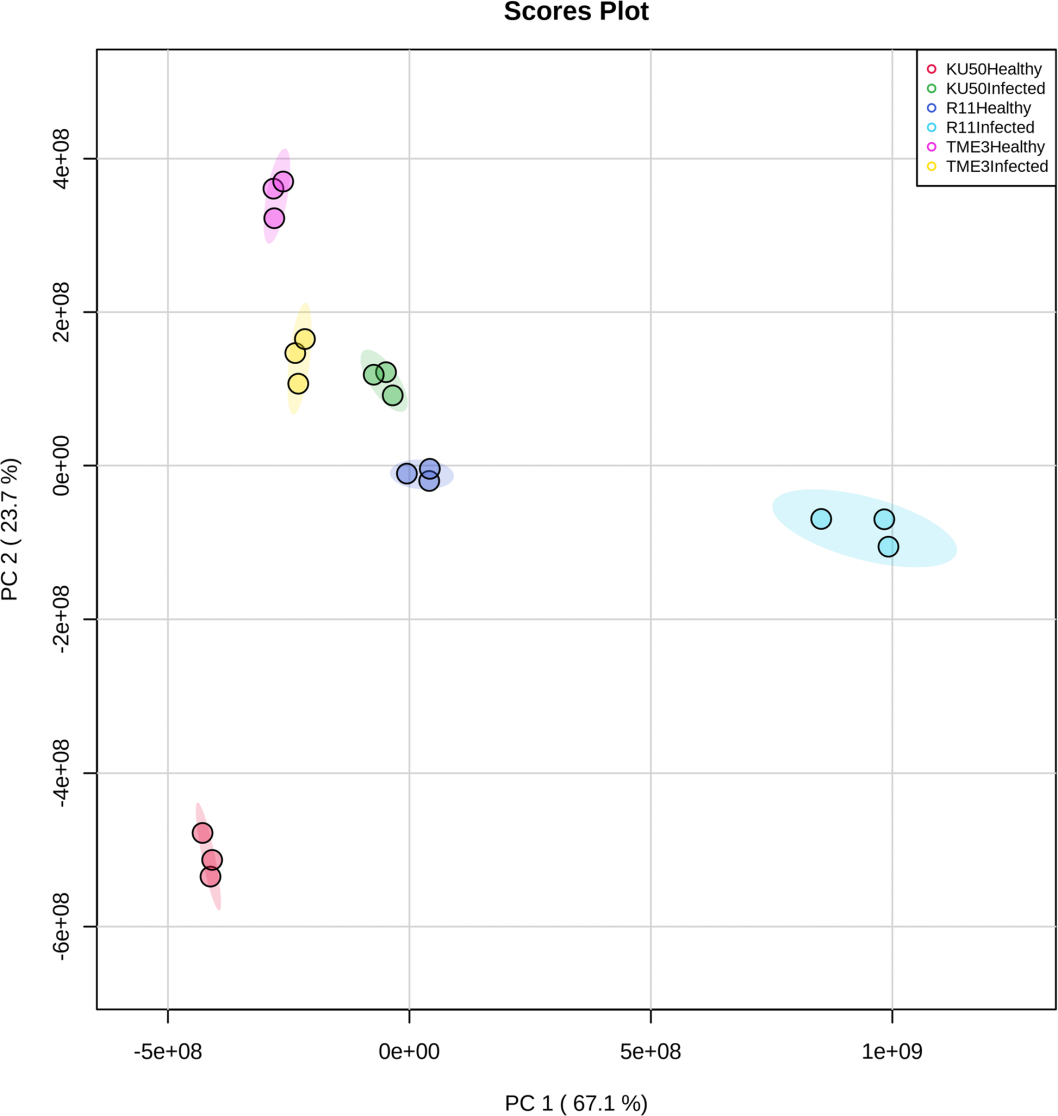
2D principal component analysis (PCA) score plot of 54 differential compounds (SLCMV-infected vs healthy groups) in all three cultivars (TME3, KU50, and R11)

Regarding the 2D PCA score plot based on the 54 compounds in the six experimental groups (Fig. 6), PC2 explained 23.7% of the variance and PC1 explained 67.1%, with clear separation of the six groups. Healthy and SLCMV-infected TME3, SLCMV-infected KU50, and healthy R11 were closely related. In contrast, SLCMV-infected R11 was separated from the other groups. Furthermore, healthy and SLCMV-infected KU50 were not closely related. During SLCMV infection, KU50 may upregulate certain metabolites as part of its defense mechanism. This reflects the genotype differences that lead to metabolite differences.

**Fig. 7:**
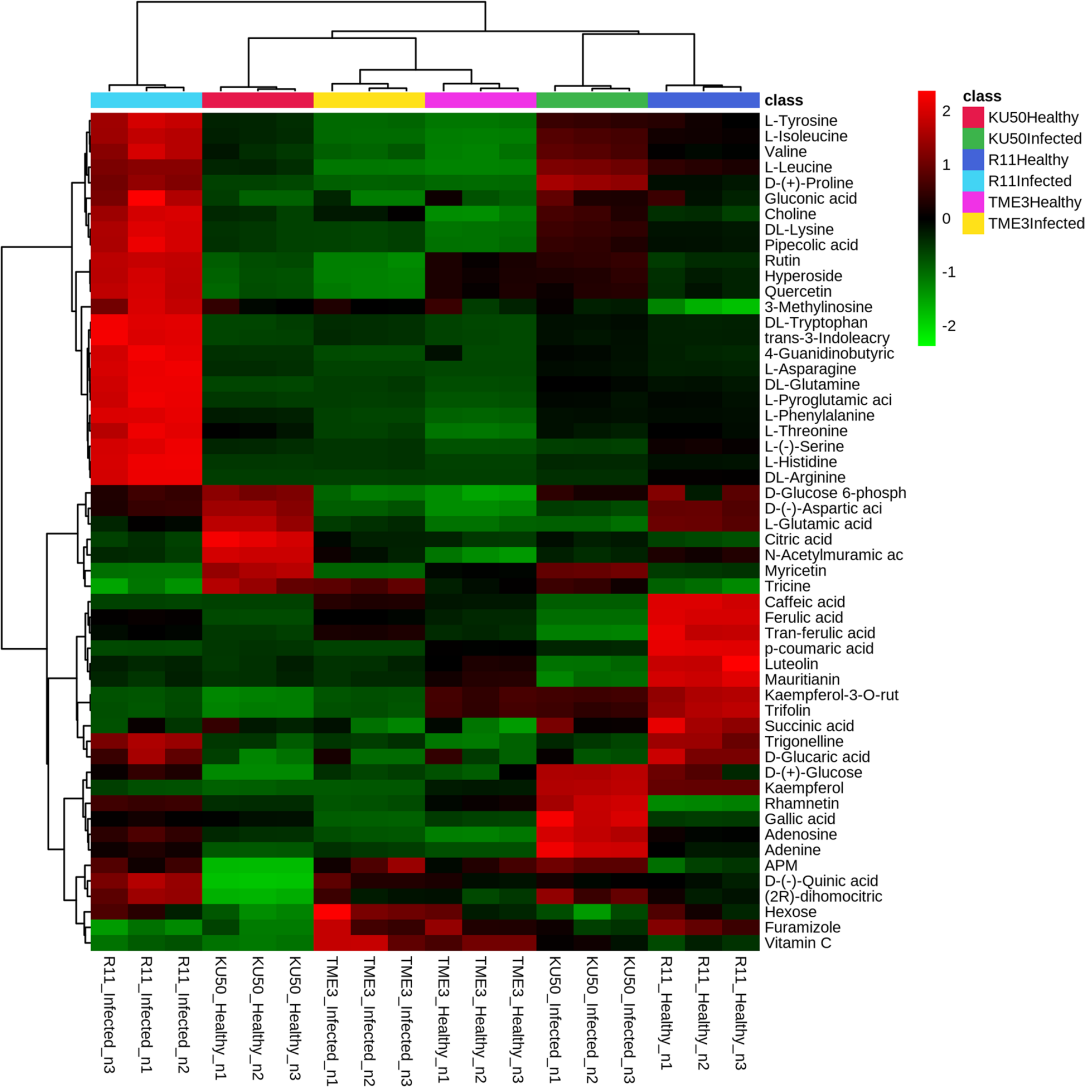
Heatmap of 54 differential compounds (SLCMV-infected vs healthy groups) in all three cultivars (TME3, KU50, and R11), based on Ward clustering method with Euclidean distance metric and one-way ANOVA followed by Fisher’s LSD test (*P* < 0.05). Red represents upregulated compounds and green represents downregulated compounds (Additional file 2)

A heatmap reflecting the intensity of the 54 co-expressed compounds among the six experimental groups is shown in Fig. 7. SLCMV-infected KU50 was related to healthy R11; however, healthy KU50 was not related to healthy or SLCMV-infected TME3. The KEGG pathway annotation of the 54 compounds is shown in Table 2.

Based on the intensity visualized in the heatmap and reference compounds in the literature, 15 key compounds [(2R)-dihomocitric acid, 3-methylinosine, APM, D-(-)-quinic acid, caffeic acid, ferulic acid, D-glucaric acid, gallic acid, DL-arginine, hyperoside, N-acetylmuramic acid, L-histidine, L-leucine, quercetin, and rutin] were selected to represent the different phenotypes in the tolerant (KU50 and TME3) and susceptible (R11) cassava cultivars and were subjected to further analysis.

### Significant differences in the 15 key differential compounds among tolerant (TME3 and KU50) and susceptible (R11) cassava cultivars

**Fig. 8:**
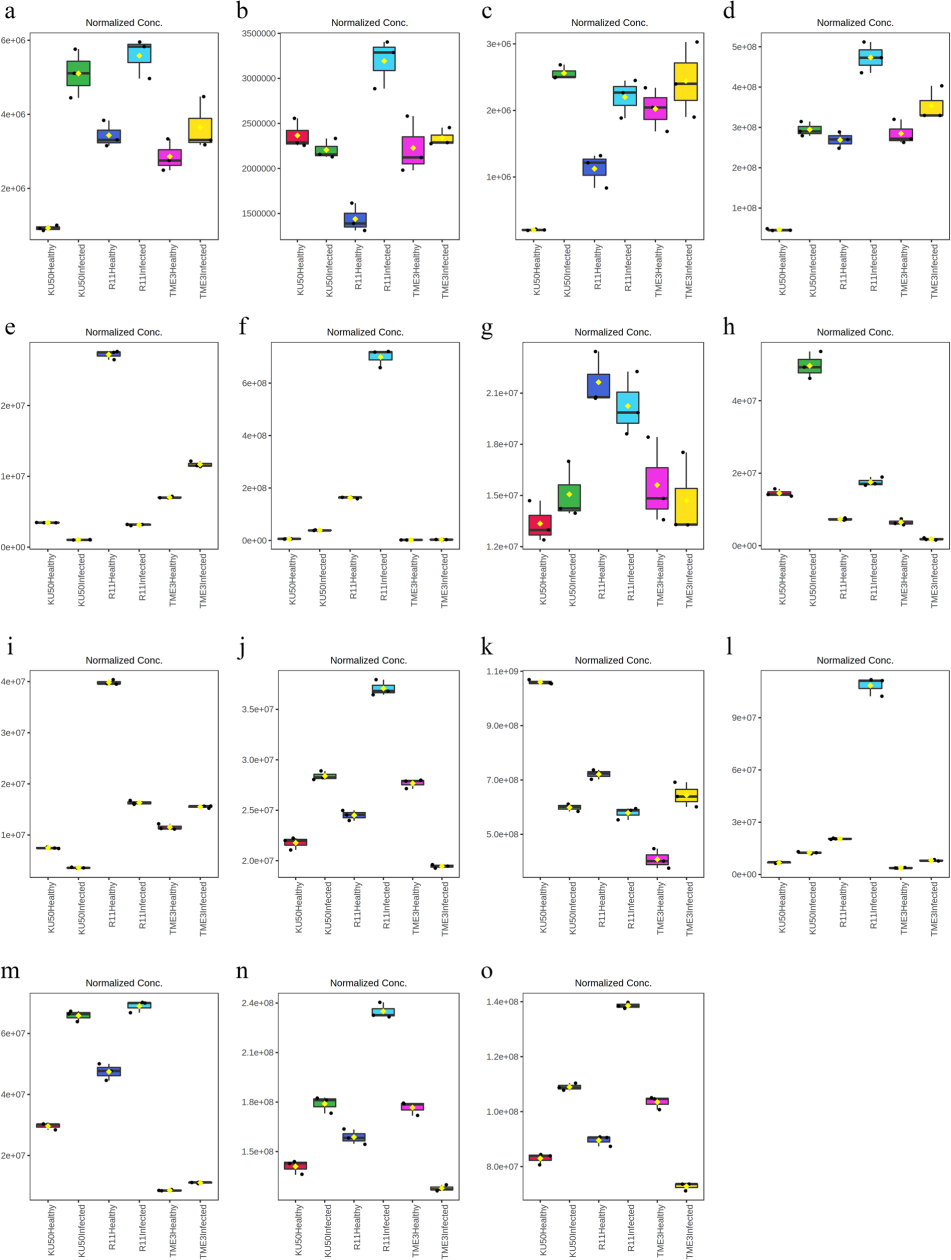
15 differential compounds (SLCMV-infected vs healthy groups), among the 54 differential compounds in all three cultivars, were tested for significant differences among tolerant (TME3 and KU50) and susceptible (R11) cassava cultivars, based on Tukey’s honestly significant difference (HSD) test (*P* < 0.05). The vertical axes were stranded for the average of detected norm. area from raw data metric and the horizontal axes were stranded for the group of three cultivars (Additional file 3). (**a**) (2R)-dihomocitric acid (**b**) 3-Methylinosine (**c**) APM (**d**) -(-)-Quinic acid (**e**) Caffeic acid (**f**) DL-Arginine (**g**) D-Glucaric acid (**h**) Gallic acid (**i**) Ferulic acid (**j**) Hyperoside (**k**) N-Acetylmuramic acid (**l**) L-Histidine (**m**) L-Leucine (**n**) Quercetin (**o**) Rutin

The 15 compounds were analyzed using Tukey’s honestly significant difference (HSD) test to identify significant differences (*p*_adj_ < 0.05) among the six experimental groups of tolerant (KU50 and TME3) and susceptible (R11) cassava cultivars (Additional file 3). The results for the 15 differential compounds (Fig. 8) can help to elucidate the differences in metabolites and metabolic pathways between the tolerant and susceptible cultivars. For example, L-histidine, which is involved in amino acid biosynthesis and ABC transporter pathways (Table 2), was present in high concentrations in R11 (highest in SLCMV-infected R11 and then healthy R11) (*p*_adj_ < 0.05) compared with the concentration in KU50 and TME3 (lowest in healthy TME3) (Fig. 8l). This was similar to the results for D-glucaric acid and DL-arginine (Fig. 8g and f). N-acetylmuramic acid, which is involved in amino sugar and nucleotide sugar metabolism and nucleotide sugar biosynthesis, including the phosphotransferase system pathway (Table 2), was present in the highest concentration in healthy KU50, but there were no significant differences among SLCMV-infected TME3, KU50, and R11 (*p*_adj_ < 0.05) (Fig. 8k). The highest concentration of L-leucine was detected in SLCMV-infected KU50 and R11, but there were low concentrations in both healthy and SLCMV-infected TME3 (Fig. 8m). The concentration of 3-methylinosine was not significantly different between KU50 and TME3 but was highest in SLCMV-infected R11 and lowest in healthy R11 (*p*_adj_ < 0.05) (Fig. 8b). Interestingly, gallic acid, which is involved in aminobenzoate degradation and phenylpropanoid biosynthesis (Table 2), was present in the highest concentration in SLCMV-infected KU50 (Fig. 8h). Quercetin, which is involved in flavone and flavanol biosynthesis (Table 2), exhibited similar concentrations in both SLCMV-infected KU50 and healthy TME3 but was highest in healthy R11 (*p*_adj_ < 0.05) (Fig. 8n). This was similar to the results for APM and D-(-)-quinic acid (Fig. 8c and d).

### Other differential compounds in tolerant (TME3 and KU50) and susceptible (R11) cultivars

Regarding the five compounds that were differentially expressed only in TME3 and KU50 (tolerant cultivars), chlorogenic acid, neochlorogenic acid, and (E)-aconitic acid were downregulated in both SLCMV-infected TME3 and KU50 (relative to their healthy counterparts), while DL-carnitine was upregulated in both SLCMV-infected TME3 and KU50 and ascorbyl glucoside was downregulated in SLCMV-infected TME3 but upregulated in SLCMV-infected KU50. 7-Hydroxycoumarine was the sole compound that was differentially expressed only in TME3 and R11, and was upregulated in SLCMV-infected TME3 but downregulated in SLCMV-infected R11. Finally, regarding the seven compounds that were differentially expressed only in KU50 and R11 (quercitrin, guanine, N-acetylornithine, uridine, vorinostat, sucrose, and lotaustralin), all were upregulated in SLCMV-infected KU50 and R11. Furthermore, sphinganine, which was only identified in TME3, was upregulated in SLCMV-infected TME3 compared with healthy TME3 (Fig. 4a).

**Fig. 9:**
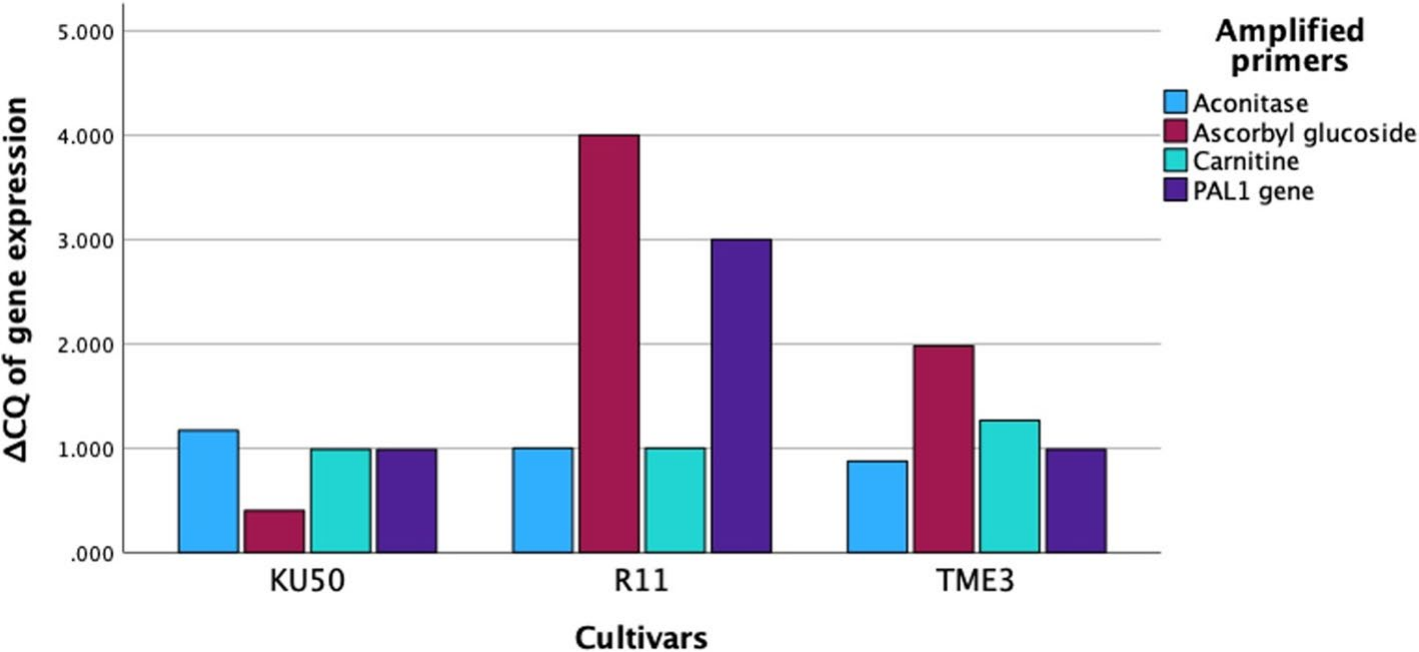
Real-time reverse-transcriptase PCR (real-time RT-PCR) data with the 2^ΔCQ^ of gene expression converted from the ΔCQ of normalized quantification cycle (C_q_) values of cDNA generated from the transcripts of total RNA in healthy and SLCMV-infected TME3, KU50, and R11 cultivars. The real-time RT-PCR data refers to the point in time that the first amplified target was detected by using specific primers for chlorogenic acid, DL-carnitine, neochlorogenic acid, (E)-aconitic acid, and ascorbyl glucoside compounds

Real-time RT-PCR was used to validate the expression of five of the metabolites including chlorogenic acid, DL-carnitine, neochlorogenic acid, (E)-aconitic acid, and ascorbyl glucoside. Using specific primers designed to amplify the genes encoding these five initial compounds, gene expression was detected in R11 as indicated by the Cq value (Fig. 9), even though there was no evidence of these five compounds being detected in R11 by the metabolomic protocol. For example, the *PAL1* gene, which leads to chlorogenic and neochlorogenic acid accumulation, was observed among all three cassava cultivars (TME3, KU50, and R11) by real-time RT-PCR. The gene expression of carnitine was highest in healthy TME3, but in cases of SLCMV infection, the highest gene expression was detected in the R11 cultivar; this conflicts with the metabolomic results, which did not detect carnitine in the R11 cultivar. Furthermore, ascorbyl glucoside, which had a high accumulation in TME3 in both conditions (healthy and SLCMV infection) in the metabolomic analysis, showed a high expression only in healthy TME3 when using amplification of the ascorbyl glucoside gene and the 2^ΔCQ^ comparison. In addition, SLCMV-infected cassava KU50 cultivar tended to have low carnitine expression in the real-time RT-PCR, but in the metabolomic analysis, a high accumulation of this compound was detected in this same condition.

## Discussion

In this study, SLCMV infection experiments were conducted on three landrace cultivars—TME3 and KU50 (tolerant cultivars) and R11 (susceptible cultivar) [36, 47]—to assess the effects of infection on plant host compounds based on UHPLC-HRMS/MS data. The study aimed to identify metabolites with key roles in tolerance vs susceptibility phenotypes (such as decreased virus titers), which could be related to the “plant recovery” hypothesis, the predominant factor in the phenomenon of tolerance [18, 20]. Cassava landrace TME3 and KU50 were selected to identify metabolites because the Department of Agriculture in Thailand [5] has recommended that Thai cassava farmers cultivate KU50 to ensure increased CMD tolerance. This is in line with a study in Thailand by Hemniem et al*.* (2019) [48] that used a grafting method involving CMD-infected stem tissue as the stock. The authors reported that R11 had CMD symptoms after 2 weeks of grafting, while KU50 and TME3 had CMD symptoms after 3 weeks. In this study, examination of phenotypic differences in the three cassava cultivars after SLCMV infection showed that the three cultivars of Thai cassava landrace exhibited significant symptomatic differences, as shown in Additional Fig. 2. Although the TME3 and KU50 cultivars are described as tolerant cultivars [48, 49], there were differences in their disease severity; the SLCMV-infected TME3 cultivar was less symptomatic in the field, followed by the SLCMV-infected KU50 cultivar, which showed moderate symptoms, while the SLCMV-infected R11 cultivar exhibited the most severe disease symptoms in field. Regarding genotypic characteristics of TME3 and KU50, Kansup et al., 2020 [50] used three different sets of molecular markers to screen for CMD resistance and revealed that the genotypes of the single nucleotide polymorphism (SNP) and expressed sequence tag (EST) makers in the peroxidase genes of TME3 and KU50 were similar, whereas those of the sequencing characterized amplified region (SCAR) and simple sequencing repeat (SSR) markers were different. These findings suggest that despite being categorized as CMD tolerant, TME3 and KU50 may have additional defense mechanisms against SLCMV that require further investigation. One omics tool, metabolomics analysis, reveals the metabolic compounds that plants synthesize in response to viruses, and this may be useful for further exploration of defense mechanisms and the basis of CMD tolerance and susceptibility.

In this study, TME3 and KU50 did not have identical levels of compounds, despite being reported to both be tolerant cultivars. For example, the L-leucine (Fig. 8m) concentration was low in both healthy and SLCMV-infected TME3, but low in healthy KU50 and high in SLCMV-infected KU50. Similar results were observed for other amino acids such as L-tyrosine, L-isoleucine, D-( +)-proline, and valine (Fig. 8). Likely, the antioxidant agents that included vitamin C and others such as ascorbic acid, ascorbyl glucoside, and ascorbyl tetraisopalmitate [51], for particularly in ascorbyl glucoside, this compound aids in protecting plants from biotic and abiotic stressors and oxidative stress [52]. Our results indicated that ascorbyl glucoside expression was downregulated in SLCMV-infected TME3 but upregulated in SLCMV-infected KU50. Remarkably, L-leucine was also high in both healthy and SLCMV-infected R11, like in SLCMV-infected KU50. Thus, L-leucine may be involved in the cassava tolerance response.

Biotic and abiotic stresses are the predominant factors that influence plant metabolite responses. In response to specific stresses, plants produce specific compounds to activate their metabolic processes to recognize, adapt to, and/or defend against stresses. Plant metabolites can be divided into primary and secondary metabolites. Primary metabolites include amino acids (glutamine, asparagine, tryptophan, lysine, histidine, proline, serine, and valine), organic acids (citrate and succinate), and sugars (trehalose) [53]. Secondary metabolites include phenolic compounds, flavonoids, 3-*trans*-caffeoylquinic (chlorogenic acid), and phenylpropanoids [54], and secondary metabolites have been shown to regulate hormone signaling [55]. A study on abiotic stress, i.e., increased temperature, reported that some secondary metabolites (such as flavonoids) were upregulated in heat-treated pericarp in citrus fruit [56].

**Fig. 10:**
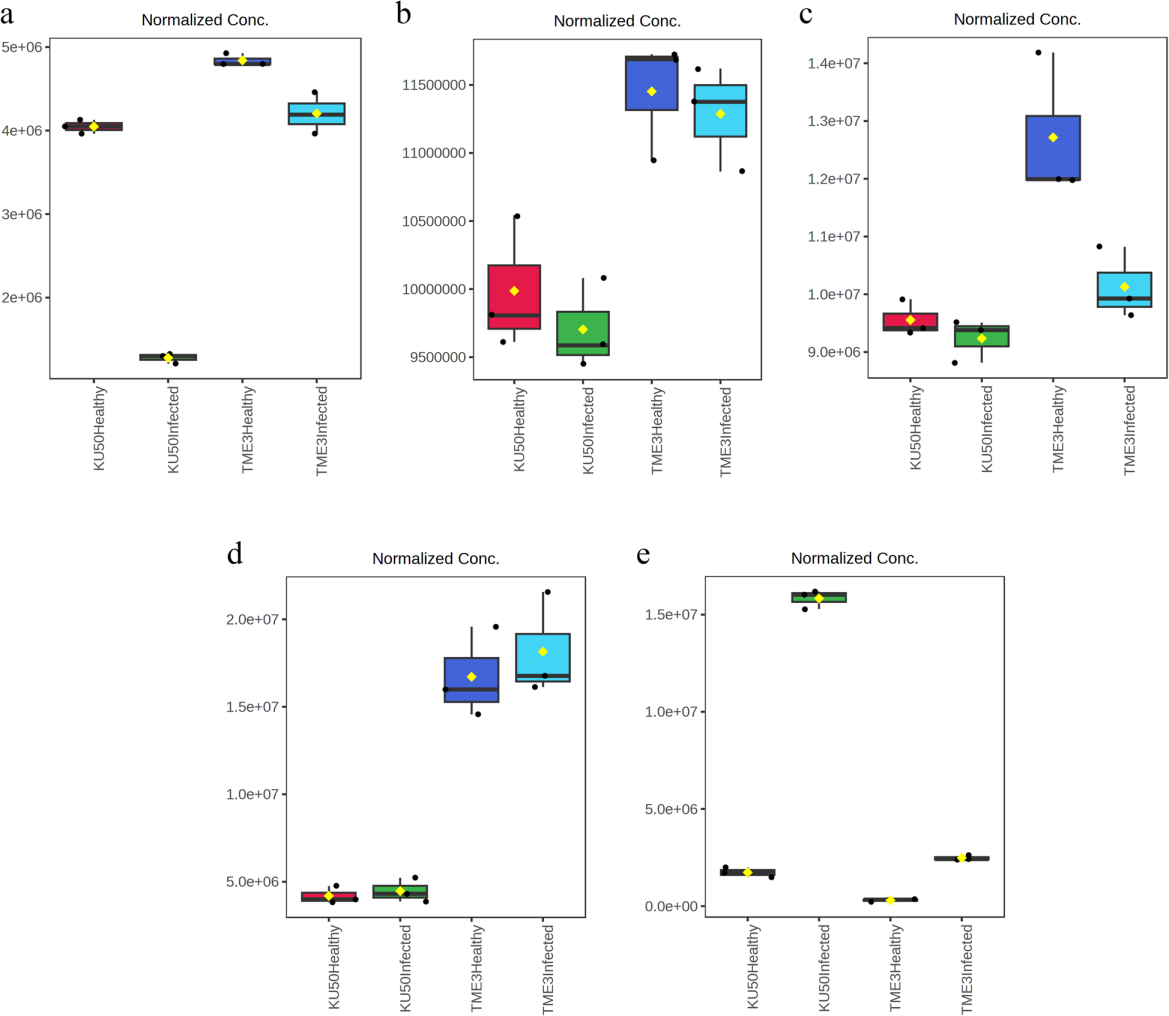
Five co-expressed compounds that were specially detected in the TME3 and KU50 cultivars and which showed similar and contrasting intensity from each other. The graph was visualized using the MetaboAnalyst planform for aligned norm. areas by adjusted Fisher’s least significant difference (LSD) test (*P* < 0.05). (**a**) Chlorogenic acid (**b**) Neochlorogenic acid (**c**) (E)-Aconitic Acid (**d**) Ascorbyl glucoside (**e**) DL-Carnitine

**Fig. 11:**
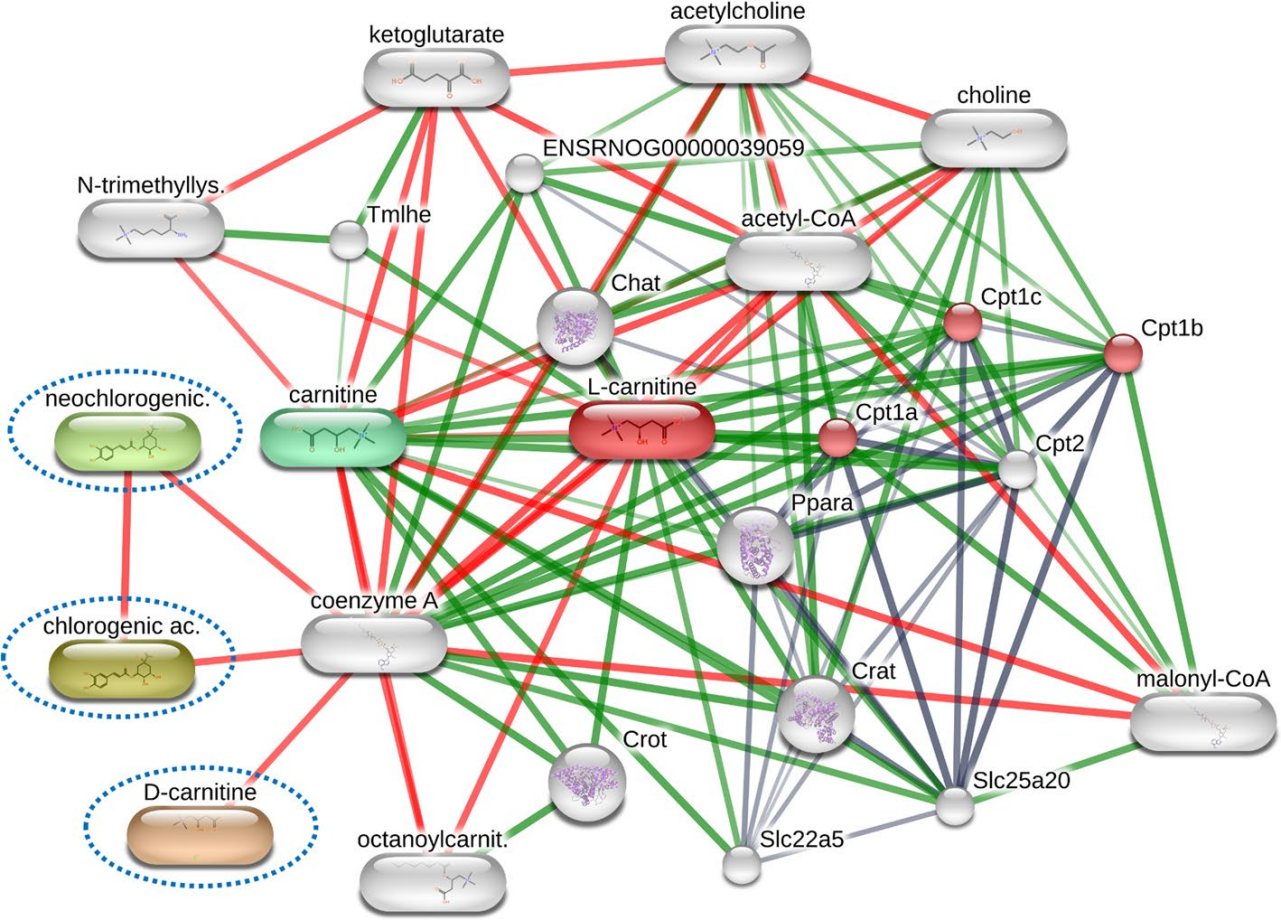
STITCH analysis within the chlorogenic acid, neochlorogenic acid, and DL-carnitine compounds presents the interaction network among these compounds and coenzyme A

Our KEGG pathway annotation of 85 differential compounds (Table 1) between SLCMV-infected vs healthy groups indicated that most compounds were involved in glucosinolate biosynthesis, purine metabolism, porphyrin and chlorophyll metabolism, flavonoid biosynthesis, aminoacyl-tRNA biosynthesis, phenylpropanoid biosynthesis, and cysteine and methionine metabolism. In analysis of the cassava response against cassava frogskin disease (CFSD) in tolerant cassava cultivars, ferulic acid, trans-caffeic acid, and neochlorogenic acid were increased, thus these metabolites may be useful markers to identify tolerance to CFSD [57]. Our metabolomic research suggests that among SLCMV-infected samples, two groups of metabolite candidates in TME3 and KU50 are upregulated and downregulated (Fig. 10). In the SLCMV infection, the TME3 and KU50 cultivars demonstrated a downregulation of chlorogenic acid, neochlorogenic acid, and aconitic acid; these substances could potentially be used as a gauge of how well the tolerant phenotype resisted biotic stresses. STITCH analysis within the chlorogenic acid, neochlorogenic acid, and DL-carnitine compounds presents the interaction network among these compounds and coenzyme A (Fig. 11). These two metabolites were reported to control the growth of various plant pathogenic fungi though the induction of reactive oxygen species (ROS) and cell apoptosis [58, 59]. Aconitic acid accumulates in sugarcane and sweet sorghum [60], and also performs a variety of biological functions within plant cells and has been noted to have anti-inflammatory, anti-fermentation, and possible nematicide properties [61]. Ascorbyl glucoside and DL-carnitine intensity were upregulated in SLCMV-infected TME3 and KU50. DL-carnitine is involved in the plant fatty acid metabolism pathway found in *Arabidopsis thaliana* and other plant species [62]. Although the DL-carnitine intensity was in the opposite direction to the accumulation of chlorogenic acid, neochlorogenic acid, and (E)-aconitic acid, these four compounds may have applications in future SLCMV marker development for tolerance phenotypes of cassava. In contrast, our real-time RT-PCR (Fig. 9) revealed that the five selected co-expressed compounds of KU50 and TME3 cultivars tended to exhibit the opposite expression to the results of the metabolomic research. Chlorogenic acid and neochlorogenic compounds were represented by amplification of the *PAL1* gene using specific primers in the real-time RT-PCR as this gene is involved with phenylpropanoid pathway activation. In SLCMV infection, the KU50 cultivar exhibited increased expression of the *PAL* gene compared with that of the TME3 cultivar using the real-time RT-PCR technique (Fig. 9). This suggests that the *PAL1* gene and chlorogenic acid compound are unrelated or that chlorogenic acid and its isomer are related to other *PAL* genes within this group, for example *PAL2* and *PAL4*, a similar trend was observed with the ascorbyl glucoside compound. Research by Padilla-González et al. (2019) [63] using metabolomic and gene expression approaches showed that during signaling, the generation of chemical compounds (secondary metabolism) in *Smallanthus sonchifolius* (locally known as yacón) of the family Asteraceae is controlled by a complex interplay between environmental conditions and developmental stage. These environmental factors influence secondary biosynthesis and may introduce the role of individual metabolic pathways, thus other genes involved in the complex signaling of secondary metabolites await discovery. In this study, metabolite interaction analysis was contracted by using STITCH analysis (Fig. 11); the interaction network presented chlorogenic acid, neochlorogenic acid, and DL-carnitine related with coenzyme A, which are involved in the PPAR signaling pathway, fatty acid degradation/metabolism, the adipocytokine signaling pathway, and the AMPK signaling pathway. There is an interesting direct interaction between neochlorogenic acid and chlorogenic acid.

There were several interesting findings related to amino acids in our study such as tricine was detected in all three experimental cultivars, but especially exhibited changes in response to SLCMV infection in TME3 and KU50, as shown in the overview heatmap (Fig. 7). Relatedly, a nontargeted GC–MS study by Peluffo et al. (2010) [64] on the fungal pathogen *Sclerotinia sclerotiorum* in susceptible and resistant *Helianthus annuus* L. (sunflower) lines reported that the amino acid isoleucine was highly associated with the susceptible sunflower line. Furthermore, in our study, the compound 7-hydroxycoumarine, which was a differential compound only in TME3 and R11, was present in a high concentration in SLCMV-infected TME3 but at a low concentration in SLCMV-infected R11 (and high in healthy R11) (Fig. 4). This differential compound is involved in coumarin biosynthesis, immune responses against *Salmonella typhimurium* in mice [65], and anti-inflammatory drug effects and neutrophil regulation in humans [66]. However, there is a lack of information about the function of 7-hydroxycoumarine in cassava. Sphinganine, which was a differential compound only in TME3, was present at a higher concentration in SLCMV-infected TME3 compared with healthy TME3. Sphinganine is involved in sphingolipid synthesis. Vicente et al. (2012) [67] reported that sphinganine was related to sphinganine-analog mycotoxins (SAMs), which disrupt the host plant and thereby facilitate necrotrophic fungi colonization, and caused sphingoid long-chain base (LCB) accumulation. This accumulation of LCB induces programmed cell death and plant immune responses, including effects in the salicylic acid pathway.

## Conclusion

In this study, we revealed differential compounds between SLCMV-infected and healthy cassava, but many unidentified compounds remain and should be explored in future research. SLCMV infection clearly affected the cassava metabolite profile. The three cultivars had unique metabolic profiles, and some of the differential compounds were present in higher concentrations in TME3 and KU50 (tolerant cultivars) than in R11 (susceptible cultivar). Such compounds may be involved in plant self-defense mechanisms, and their differences among the three SLCMV-infected cultivars support the “phenotypic variation” theory. The differential compounds could be used as markers to classify SLCMV-infected cultivars such as TME3, KU50, and R11, as they may underlie the phenotype differences among the cultivars.

Plant–virus interactions, including cassava–SLCMV interactions, are very complex. Many details of plant–virus interactions require further investigation; however, many factors can influence the results owing to the complexity of the processes, such as compound detection methods (including selecting a suitable analysis platform), compound identification methods, and methods to identify the associated pathways. In the future, the differential metabolic profiles of healthy and SLCMV-infected cultivars may help to develop more knowledge, tools, and technologies and eventually identify a tolerant genotype involving cassava CMD-tolerance markers, which will facilitate the development of new cultivars of this important crop.

## Methods

### Sample collection

Cassava landrace TME3, KU50, and R11 cultivars with infected and healthy (non-infected) stems were vegetatively propagated. Next, the stems were planted in a greenhouse of the Department of Plant Pathology, Faculty of Kasetsart University, Thailand. The stems were cut into 15-cm pieces, each with 3–4 buds, and 18 were planted in soil in 8-inch-diameter plastic pots. When the 18 plants (3 cultivars × 2 treatments × 3 repeats) were 45 days old, the leaves below the apex were collected. The leaves from each set of 3 repeats were pooled into one sample. The leaves were transferred to a laboratory using polybags and immediately placed into vials for polymerase chain reaction (PCR) and ultra-high-performance liquid chromatography high-resolution mass spectrometry (UHPLC-HRMS/MS).

### CMD detection by PCR

The DNA of cassava leaves was extracted using the cetyltrimethylammonium bromide (CTAB) method [68]. Briefly, cassava leaves were ground into fine powder in liquid nitrogen, then 20 mg powder was mixed with 700 µL CTAB buffer and incubated at 65 °C for 30 min. Thereafter, 700 µL chloroform:isoamyl alcohol (24:1) was added. For DNA precipitation, 700 µL isopropanol alcohol was added and the mixture was incubated at -20 °C for 3 h. The DNA pellet was cleaned twice with 70% ethanol and dried at room temperature. The pellet was then resuspended in ddH_2_O containing 100 µg/mL RNase (Thermo Fisher Scientific, Waltham, MA, USA) and stored at -20 °C.

The quality and quantity of the DNA samples were assessed by gel electrophoresis on a 1.5% agarose TAE gel containing RedSafe Nucleic Acid Staining Solution (iNtRON Biotechnology, Sangdaewon, South Korea) in 0.5 × TAE buffer (1 M Tris HCI pH 8, 0.5 M ethylenediaminetetraacetic acid [EDTA], and glacial acetic acid at 100 V for 30 min. A 1 kb DNA ladder (Thermo Scientific, USA) was used as the reference. The results were analyzed using SYNGENE software (Synoptics Ltd., Cambridge, UK). A NanoDrop spectrophotometer (NanoDrop Technologies, Thermo Scientific) was used to confirm the quantity and purity of the DNA.

PCR was used to detect the *AV1* gene of SLCMV. Forward (5ʹ-GTT GAA GGT ACT TAT TCC C-3ʹ) and reverse (5ʹ-TAT TAA TAC GGT TGT AAA CGC-3ʹ) primers were used to amplify the partial *AV1* gene fragment referred to in a protocol described by Saokham et al*.* (2021) [69]. The 25-µL reaction contained 1 × PCR buffer (PCR Biosystems, London, UK), 0.2 µM each of forward and reverse primers, and 50 ng genomic DNA. The thermal cycling conditions for amplification were initial denaturation at 94 °C for 5 min; 35 cycles of denaturation at 94 °C for 40 s, annealing at 55 °C for 40 s, and elongation at 72 °C for 40 s; and then a final elongation at 72 °C for 5 min. The PCR products underwent electrophoresis on 1.5% agarose TAE gel containing RedSafe Nucleic Acid Staining Solution (iNtRON Biotechnology).

### RNA extraction, cDNA library construction, and real-time PCR

Healthy and diseased cassava leaves of TME3 and KU50 cultivars were collected and immediately frozen in liquid nitrogen then stored at -80 °C until use. RNA was extracted from the leaves by using a RNeasy Mini Kit (Qiagen, Hilden, Germany). For cDNA library construction, cDNA was synthesized from the total RNAs by using reverse transcriptase (Thermo Fisher Scientific, Waltham, MA, USA). Specific primers were designed from the literature review of related genes involved in the chlorogenic acid, DL-carnitine, neochlorogenic acid, (E)-aconitic acid, and ascorbyl glucoside metabolic pathway (Additional file 4). The real-time PCR mixture was prepared by using 5 µL qPCRBIO SyGreen Mix Lo-ROX—100 rxns (COPENHAGEN BIOTECH SUPPLY, Denmark), 0.5 µL each of forward and reverse primers, 3 µL nuclease-free water, and 1 µL cDNA template (before use as templates, the cDNA concentration was adjusted by diluting to 100 ng/mL in all samples). Ubiquitin housekeeping gene was used to normalize Cq data. Real-time PCR was performed by CFX96 Real-Time PCR Detection (Bio Rad, CA, USA). Subsequently, the detected quantification cycle (Cq) values were used to calculate 2^ΔCQ^ (normalized Cq of diseased samples – normalized Cq of healthy samples). The relative expression between genes and both tolerant cultivars as a condition of healthy and SLCMV infection were then calculated from all RT-PCR data using ΔCQ (raw data were attached in Additional file 4).

### Metabolite profiling by UHPLC-HRMS/MS

A previously described method from Nehela et al. (2016) [70] was used to extract metabolites from 100 mg fresh cassava leaves per sample. The leaves were ground using a glass rod and the metabolites were extracted with 750 µL solvent comprising 75% methanol, 20% water, and 5% formic acid (FA). The mixture was vortexed for 30 s, incubated on ice for 10 min, and centrifuged at 1,500 g for 5 min. The supernatant was collected, and the pellet underwent the extraction process two more times. The supernatants were combined and dried using a centrifugal concentrator.

Next, 200 ppm cassava metabolite extract (2 µL) was separated on a Hypersil GOLD™ Vanquish C18 column (2.1 × 100 mm, 1.9 µm, Thermo Scientific) with a guard column at 40 °C and a flow rate of 0.4 mL/min. Mobile phase A comprised 0.1% FA in water and mobile phase B comprised 0.1% FA in acetonitrile. After 4 min at 5% B, the percentage was increased to 90% B over 10 min. The column was then flushed with 90% B for 4 min and decreased to 5% B over 1 min before returning to the starting condition for 25 min.

MS acquisition was performed using a Q-Exactive HF-X Orbitrap mass spectrophotometer (Thermo Scientific) and a heated electrospray ionization (HESI) ion source. Negative and positive ions were detected using full-scan MS1/data-dependent MS2 (dd-MS2) mode, with the following settings: spray voltage, 3.5 kV (positive) and 2.5 kV (negative); sheath gas, 45 AU; auxiliary gas, 10 AU; sweep gas, 2 AU; capillary temperature, 250 °C; full-scan MS1 resolution, 120,000; ddMS2 resolution, 30,000; scan range, 100–1500 m/z; automatic gain control target, 3e6; maximum injection time, 100 ms; and stepped N(CE) at 20, 30, and 40 eV. The acquired files were processed using Compound Discoverer software, and the mzCloud, mzVault, and ChemSpider databases were used for annotation.

### Data analysis

To analyze the levels of compounds (including peak levels) in each sample, the MS data were normalized and placed in comma separated values (.csv) files, with adjustment to ensure correct column alignment. One-way analysis of variance (ANOVA) followed by Fisher’s least significant difference (LSD) test (*P* < 0.05) were used to identify differential compounds (by comparing the healthy vs SLCMV-infected groups in particular cultivars) in the MetaboAnalyst platform. The data metric then qualitied by using an interquartile range (IQR) filtered features adjusted 25% in standard deviation QC samples; this mentioned relative standard deviation was found to be acceptable, and also enabled the detection of values variables throughout the experiment condition by using planform alignment. Heatmaps, 2D PCA scores plots, and hierarchical clustering dendrograms were explored by MetaboAnalyst then used to identify simplified relationships between the three cultivars (TME3, KU50, and R11) and between healthy and SLCMV-infected groups within each cultivar. Heatmaps, using adjusted *P*-values < 0.05 from the Fisher’s LSD test with one-way ANOVA, were based on the Ward clustering method with Euclidean distance metric for differentially expressed metabolite datasets in susceptible and tolerant cultivars. The groups were clustered by using Euclidean distance to calculate a distance metric between the groups and using the Ward clustering algorithm, and the results were then visualized in a dendrogram. A Venn diagram of the differential compounds was constructed using jvenn (http://jvenn.toulouse.inra.fr/app/index.html; [71]) to visualize the differential compounds shared among the groups.

To identify the functions and pathways associated with the differential compounds, KEGG (https://www.genome.jp/kegg/) [72] pathway analysis was performed, based on *Arabidopsis thaliana* (model organism) and *Manihot esculenta* (cassava), which included annotation using the literature and PubChem database (https://pubchem.ncbi.nlm.nih.gov/).

Using the associations shown in the heatmap and reference compounds reported in the literature, 15 key compounds were selected that represented the different phenotypes in the tolerant (KU50 and TME3) and susceptible (R11) cultivars. The 15 compounds were then analyzed in Statistical Tool for Agricultural Research (STAR) 2.0.1 (http://bbi.irri.org/products) using Tukey’s honestly significant difference (HSD) test to identify significant differences (*p*_adj_ < 0.05) among the six experimental groups of tolerant (KU50 and TME3) and susceptible (R11) cultivars. The KEGG pathways associated with these 15 key compounds were also investigated.

## Supplementary Information

**Additional file 1. **Raw UHPLC-HRMS/MS data for 85 compounds (this file separates into three subfiles based on cultivar).

**Additional file 2. **Raw UHPLC-HRMS/MS data for 54 shared compounds that were differential compounds in all three cultivars (TME3, KU50, and R11), including results based on one-way ANOVA followed by Fisher’s LSD test (*P* < 0.05) in MetaboAnalyst.

**Additional file 3. **Raw UHPLC-HRMS/MS data for 15 differential compounds that were tested for significant differences among tolerant (TME3 and KU50) and susceptible (R11) cultivars, based on Tukey’s honestly significant difference (HSD) tests (*p*_adj_ < 0.05) in STAR 2.0.1.

**Additional file 4. **Raw data of specific primers and sequence data that were used to detect amplification targets from real-time RT-PCR based on chlorogenic acid, DL-carnitine, neochlorogenic acid, (E)-aconitic acid, and ascorbyl glucoside compounds, with reference to other literature reviews.

**Additional file 5: Figure 1.** PCR confirmation of SLCMV-infected and healthy cassava samples. Lane 1: 1 kb DNA ladder marker (Thermo Scientific, USA), lane 2: positive control (DNA product from extracted SLCMV-infected cassava), lane 3: negative control (DNA product from extracted healthy cassava), lane 4: SLCMV-infected TME3 cultivar, lane 5: SLCMV-infected KU50 cultivar, lane 6: SLCMV-infected R11 cultivar, lane 7: healthy TME3 cultivar, lane 8: healthy KU50 cultivar, and lane 9: healthy R11 cultivar.

**Additional file 6: Figure 2.** SLCMV-infected and healthy cassava in the Thai’s cassava by our field survey, especially in tolerant cultivars. a: SLCMV-infected TME3 cultivar, b: SLCMV-infected KU50 cultivar, c: SLCMV-infected R11 cultivar.

## Data Availability

The data sets supporting the conclusions of this article are included with the article and its additional files.
