# Supplementary figures and images for: Metabolic profiles of Sri Lankan cassava mosaic virus-infected and healthy cassava (Manihot esculenta Crantz) cultivars with tolerance and susceptibility phenotypes
Source: BMC Plant Biol. 2023 Apr 5;23:178. doi: 10.1186/s12870-023-04181-3 (PMC10074701; doi:10.1186/s12870-023-04181-3)

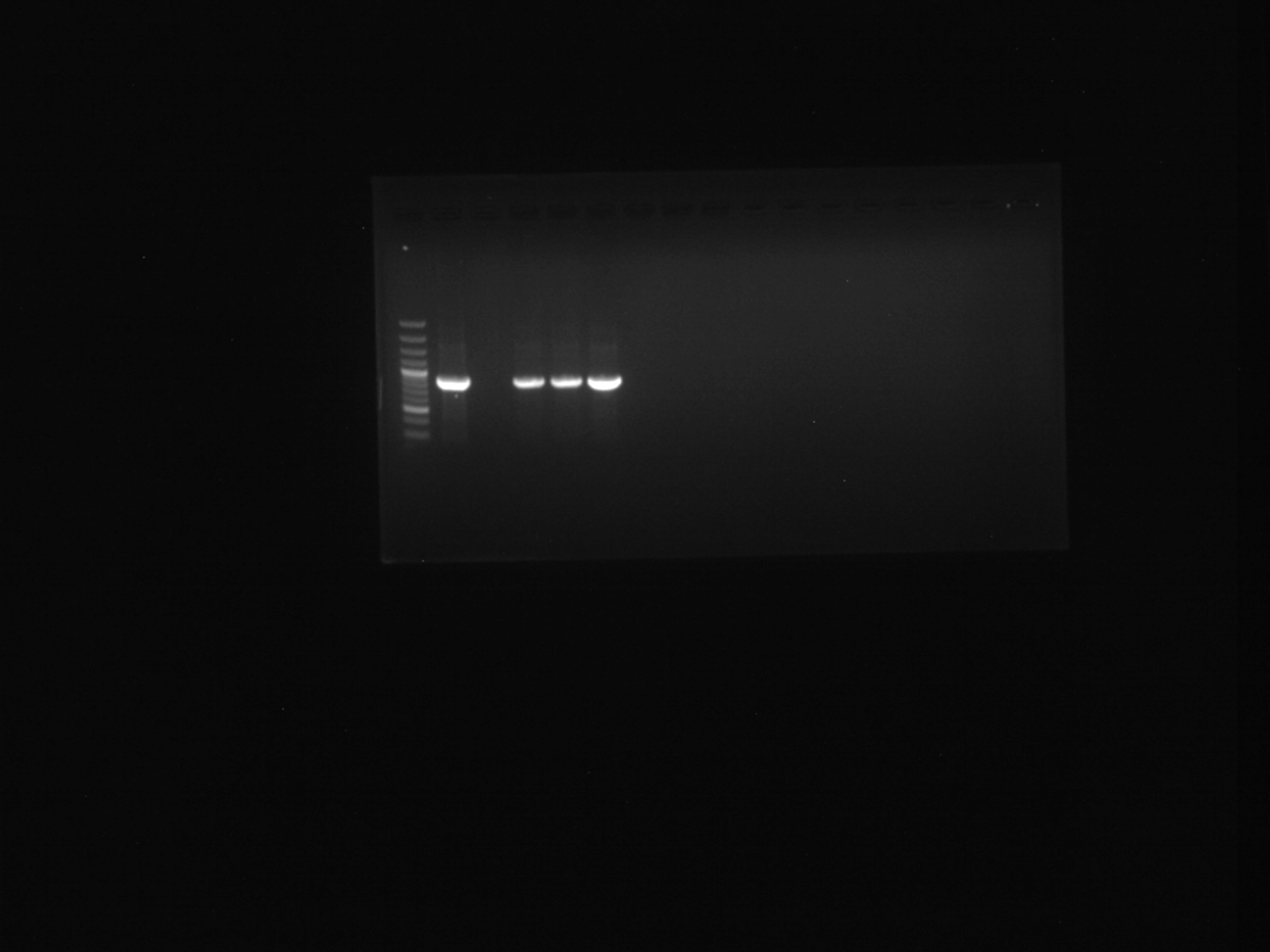

Supplement: Supplementary file 5 — Additional file 5: Figure 1. PCR confirmation of SLCMV-infected and healthy cassava samples. Lane 1: 1 kb DNA ladder marker (Thermo Scientific, USA), lane 2: positive control (DNA product from extracted SLCMV-infected cassava), lane 3: negative control (DNA product from extracted healthy cassava), lane 4: SLCMV-infected TME3 cultivar, lane 5: SLCMV-infected KU50 cultivar, lane 6: SLCMV-infected R11 cultivar, lane 7: healthy TME3 cultivar, lane 8: healthy KU50 cultivar, and lane 9: healthy R11 cultivar. [file 12870_2023_4181_MOESM5_ESM.tif]

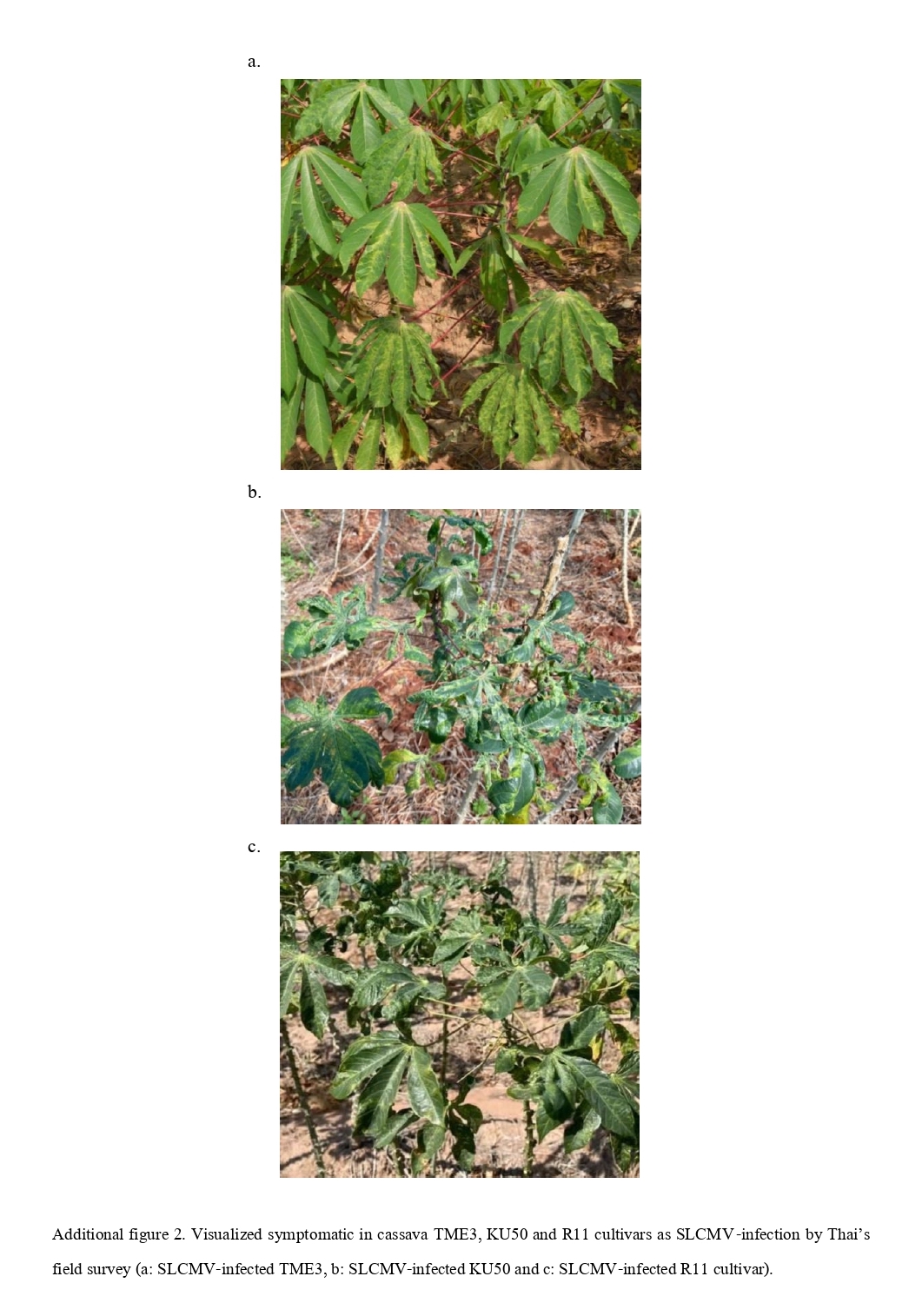

Supplement: Supplementary file 6 — Additional file 6: Figure 2. SLCMV-infected and healthy cassava in the Thai’s cassava by our field survey, especially in tolerant cultivars. a: SLCMV-infected TME3 cultivar, b: SLCMV-infected KU50 cultivar, c: SLCMV-infected R11 cultivar. [file 12870_2023_4181_MOESM6_ESM.jpg]
